# Cytotoxic and Anti-Inflammatory Metabolites from the Soft Coral *Scleronephthya gracillimum*

**DOI:** 10.3390/md11061853

**Published:** 2013-05-29

**Authors:** Hui-Yu Fang, Chi-Hsin Hsu, Chih-Hua Chao, Zhi-Hong Wen, Yang-Chang Wu, Chang-Feng Dai, Jyh-Horng Sheu

**Affiliations:** 1 Department of Marine Biotechnology and Resources, National Sun Yat-sen University, Kaohsiung 80424, Taiwan; E-Mails: ahui0220@yahoo.com.tw (H.-Y.F.); hsuch@mail.nsysu.edu.tw (C.-H.H.); chaochihhua@hotmail.com (C.-H.C.); wzh@mail.nsysu.edu.tw (Z.-H.W.); 2 School of Pharmacy, China Medical University, Taichung 40402, Taiwan; E-Mail: yachwu@mail.cmu.edu.tw; 3 Chinese Medicine Research and Development Center, China Medical University Hospital, Taichung 40402, Taiwan; 4 Asia-Pacific Ocean Research Center, National Sun Yat-sen University, Kaohsiung 80424, Taiwan; 5 Center for Molecular Medicine, China Medical University Hospital, Taichung 40402, Taiwan; 6 Institute of Oceanography, National Taiwan University, Taipei 10617, Taiwan; E-Mail: corallab@ntu.edu.tw; 7 Graduate Institute of Natural Products, Kaohsiung Medical University, Kaohsiung 80708, Taiwan

**Keywords:** soft coral, *Scleronephthya gracillimum*, cytotoxicity activity, anti-inflammatory activity

## Abstract

Five new pregnane-type steroids, sclerosteroids J–N (**1**–**5**), and a diterpenoid with a new chemotype 3-methyl-5-(10′-acetoxy-2′,6′,10′-trimethylundecyl)-2-penten-5-olide (**6**), have been isolated from a soft coral *Scleronephthya gracillimum*. The structures of the metabolites were determined by extensive spectroscopic analysis. Compound **4** exhibited cytotoxicity against HepG2, A549, and MDA-MB-231 cancer cell lines. Furthermore, steroids **2** and **4** were found to significantly inhibit the accumulation of the pro-inflammatory iNOS protein, and **1**, **2**, **4** and **5** could effectively reduce the accumulation of COX-2 protein in LPS-stimulated RAW264.7 macrophage cells.

## 1. Introduction

**Chart 1:**
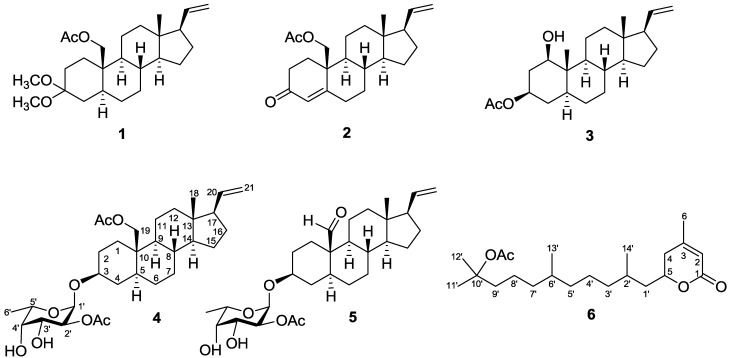
Structures of metabolites **1**–**6**.

Soft corals have proven to be rich sources of terpenoids [1]. In previous studies, a series of novel secondary metabolites, including two alkyl glycerol ethers, one cembrane-based diterpenoid, one indole alkaoid and pregnane-type steroids have been isolated from the soft corals of the genus *Scleronephthya* [2,3,4,5,6,7]. During the course of search for bioactive metabolites from marine invertebrates, several pregnane-type compounds also have been isolated from soft corals, such as *Carijoa* sp. [8], *Dendronephthya griffini* [9], *Dendronephthya* sp. [10], *Eunicella cavolini* [11], *Eunicella verrucosa* [12], *Muricea austera* [13], *Stereonephthya crystallina* [14], and *Subergorgia suberosa* [15]. Our recent study of the chemical constituents of the Green Island soft coral *Scleronephthya* *gracillimum* [7] has yielded sclerosteroids A–I, of which some were found to exhibit significant cytotoxicity and anti-inflammatory activities. Our continuing chemical investigation on the same collection of this organism, with the aim of discovering other biologically active natural products, again led to the isolation of five new sclerosteroids J–N (**1**–**5**), and a new type of diterpenoidal δ-lactone, 3-methyl-5-(10′-acetoxy-2′,6′,10′-trimethylundecyl)-2-penten-5-olide (**6**). (Chart 1). The structures of **1**–**6** have been established by extensive spectroscopic analysis, including 2D NMR (^1^H–^1^H COSY, HMQC, HMBC, and NOESY) correlations. The cytotoxicity of compounds **1**–**6** against human liver carcinoma (HepG2), human lung carcinoma (A-549), and human breast carcinoma (MDA-MB-231) cell lines was measured. The ability of **1**, **2**, and **4**–**6** to inhibit the up-regulation of pro-inflammatory iNOS (inducible nitric oxide synthase) and COX-2 (cyclooxygenase-2) proteins in LPS (lipopolysaccharide)-stimulated RAW264.7 macrophage cells also was evaluated.

## 2. Results and Discussion

**Figure 1:**
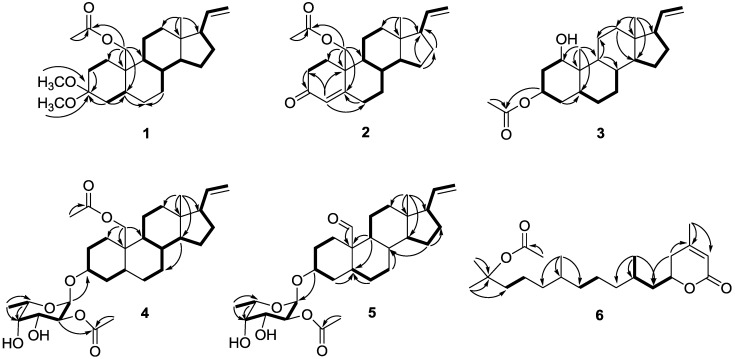
^1^H–^1^H COSY (▬) and HMBC (→)correlations for **1**–**6**.

**Figure 2:**
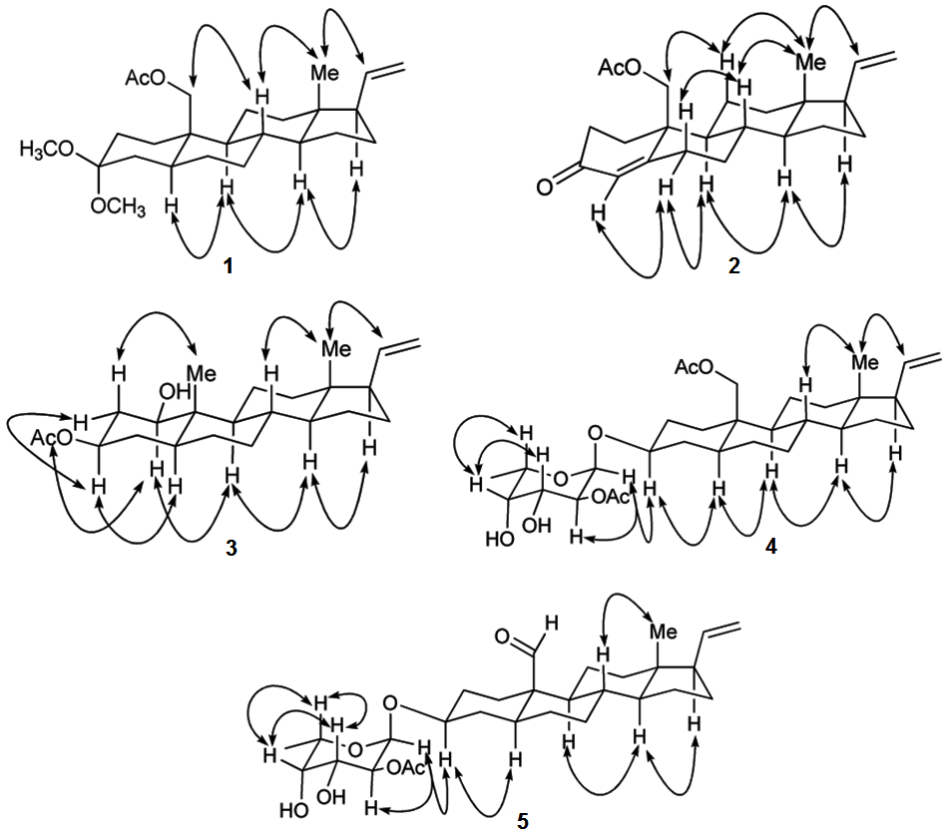
Key NOESY Correlations for **1**–**5**.

Sclerosteroid J (**1**) was obtained as colorless oil. The HRESIMS of **1** showed a pseudomolecular ion peak at *m/z* 427.2827 [M + Na]^+^, consistent with a molecular formula of C_25_H_40_O_4_ and requiring six degrees of unsaturation. The IR spectrum revealed the presence of ester-carbonyl group (1739 cm^−1^) in **1**. The ^13^C NMR and DEPT spectroscopic data (Table 1) showed the presence of 25 carbon signals, including 4 methyls, 10 sp^3^ methylenes, 5 sp^3^ methines, 1 sp^2^ methine, 1 sp^2^ methylene, 1 sp^2^ quaternary and 3 sp^3^ quaternary carbons. The ^1^H NMR showed the presence of a tertiary methyl at δ_H_ 0.57 (3H, s), two methoxyls at δ_H_ 3.15 (3H, s), and 3.19 (3H, s), an acetoxymethyl at δ_H_ 4.16 (1H, d, *J* = 12.0 Hz), 4.35 (1H, d, *J* = 12.0 Hz), and 2.05 (3H, s), and a vinyl group at δ_H_ 4.96 (1H, br d, *J* = 17.0 Hz), 4.97 (1H, br d, *J* = 10.5 Hz), and 5.74 (1H, ddd, *J* = 17.0, 10.5, 7.5 Hz). These spectroscopic data showed that **1** might be a pregnane with an acetoxymethyl substituent at C-10 on the basis of the disappearance of an H_3_-19 singlet around δ_H_ 0.80–1.10 and the presence of an AB doublet at δ_H_ 4.16 (*J =* 12.0 Hz), and 4.35 (*J =* 12.0 Hz). The molecular skeleton of **1** was determined by ^1^H–^1^H COSY and HMBC correlations as shown in Figure 1, in which C-3 (δ_C_ 100.0) was HMBC correlated by protons of two methoxy groups and CH_2_-2. Thus, similar to known compound 11α-acetoxy-3,3-dimethoxy-5α-pregn-20-ene [11], **1** also has two methoxyl substituents at C-3. The presence of an sp^3^ methylene substituent at C-19 was further confirmed by the HMBC correlations from H_2_-19 to C-1, C-5, C-9 and C-10. The relative stereochemistry of **1** was determined by correlations of a 2D NOE experiment (Figure 2). The observed NOE correlations between H-20 and H_3_-18, H_3_-18 and H-8, H-8 and H_2_-19, H-17 and H-14, H-14 and H-9, and H-9 and H-5 revealed the β-orientation of H-8, H_3_-18, H_2_-19 and H-20, and α-orientation of H-5, H-9, H-14 and H-17. On the basis of the above spectroscopic data, the structure of sclerosteroid J (**1**) was established as 19-acetoxy-3,3-dimethoxy-5α-pregn-20-ene.

**Table 1:** ^1^H and ^13^C NMR spectroscopicdata of**1**–**3**.

Position		1		2		3	
		δ_H_ (*J* in Hz) ^a^	δ_C_ (mult.) ^b^	δ_H_ (*J* in Hz) ^a^	δ_C_ (mult.) ^b^	δ_H_ (*J* in Hz) ^a^	δ_C_ (mult.) ^b^
1	α	1.01 m	29.7, CH_2_^c^	1.84 m	33.7, CH_2_	3.51 td (11.5, 5.0) ^d^	76.8, CH
	β	2.05 m		2.34 m			
2	α	1.36 m	28.4, CH_2_	2.35 m	34.7, CH_2_	2.04 m	38.1, CH_2_
	β	1.92 m		2.61 m		1.53 m	
3			100.0, C		199.5, C	4.70 tt (11.5, 5.0)	70.2, CH
4	α	1.42 m	35.8, CH_2_	5.91d (1.5)	126.7, CH	1.33 m	34.0, CH_2_
	β	1.70 m				1.56 m	
5		1.46 m	42.7, CH		166.0, C	1.08 m	42.2, CH
6	α	1.26 m	28.0, CH_2_	2.34 m	33.2, CH_2_	1.37 m	28.3, CH_2_
	β			2.41 m			
7	α	0.97 m	31.9, CH_2_	1.08 m	32.3, CH_2_	1.67 m	32.0, CH_2_
	β	1.73 m		1.91 m		0.87 m	
8		1.49 m	35.9, CH	1.62 m	36.3, CH	1.34 m	36.0, CH
9		0.84 m	54.3, CH	1.10 m	54.4, CH	0.88 m	54.9, CH
10			38.3, C		41.9, C		41.6, C
11	α	1.63 m	21.8, CH_2_	1.67 m	21.2, CH_2_	1.42 m	24.1, CH_2_
	β	1.30 m		1.41 m		2.07 m	
12	α	0.97 m	37.9, CH_2_	1.03 m	37.5, CH_2_	1.06 m	37.8, CH_2_
	β	1.65 m		1.73 m		1.68 m	
13			43.6, C		43.4, C		43.0, C
14		1.00 m	55.9, CH	1.00 m	55.4, CH	0.99 m	55.5, CH
15	α	1.67 m	24.7, CH_2_	1.70 m	24.6, CH_2_	1.66 m	25.1, CH_2_
	β	1.17 m		1.23 m		1.14 m	
16	α	1.77 m	27.2, CH_2_	1.81 m	27.1, CH_2_	1.74 m	27.0, CH_2_
	β	1.54 m		1.58 m		1.53 m	
17		1.93 m	55.3, CH	1.96 m	55.1, CH	1.93 m	55.5, CH
18		0.57 s	13.0, CH_3_	0.64 s	12.9, CH_3_	0.58 s	12.8, CH_3_
19		4.16 d (12.0) ^d^	62.5, CH_2_	4.17 d (11.0)	67.0, CH_2_	0.87 s	6.7, CH_3_
		4.35 d (12.0)		4.67 d (11.0)			
20		5.74 ddd (17.0, 10.5, 7.5)	140.0, CH	5.74 ddd (17.0, 11.0, 6.5)	139.2, CH	5.75 ddd (17.0, 11.0, 7.5)	139.8, CH
21		4.96 br d (17.0)	114.5, CH_2_	4.98 br d (17.0)	114.9, CH_2_	4.95 br d (17.0)	114.4, CH_2_
		4.97 br d (10.5)		4.99 br d (11.0)		4.96 br d (11.0)	
OH						1.25 d (11.5)	
OAc		2.05s	21.2, CH_3_	2.00 s	21.0, CH_3_	2.02 s	21.3, CH_3_
			171.2, C		170.7, C		170.6, C
OMe		3.15 s	47.5, CH_3_				
		3.19 s	47.6, CH_3_				

^a^ Spectra recorded at 500 MHz in CDCl_3_; ^b^ Spectra recorded at 125 MHz in CDCl_3_; ^c^ Deduced from DEPT; ^d^ *J* values (Hz) in parentheses.

Sclerosteroid K (**2**) has a molecular formula of C_23_H_3__2_O_3_ as determined by HRESIMS, appropriate for eight degrees of unsaturation. The ^1^H and ^13^C NMR, including DEPT spectrum, exhibited the presence of a tertiary methyl (δ_H_ 0.64, 3H, s; δ_C_ 12.9), a primary acetoxymethyl group (δ_H_ 4.17, 1H, d, *J* = 11.0 Hz; 4.67, 1H, d, *J* = 11.0 Hz; δ_C_ 67.0), a vinyl group (δ_H_ 4.98, 1H, br d, *J* = 17.0 Hz; 4.99, 1H, br d, *J* = 11.0 Hz; 5.74, 1H, ddd, *J* = 17.0, 11.0, 6.5 Hz; δ_C_ 114.9, 139.2), and an enone group (δ_H_ 5.91, 1H, d, *J* = 1.5 Hz; δ_C_ 126.7, 166.0, 199.5) (Table 1). These spectroscopic data showed that **2** might have a 4,20-dien-3-one-pregnane skeleton [16,17] with an acetoxymethyl substituent at C-10 on the basis of the disappearance of an H_3_-19 singlet around δ_H_ 0.80–1.10 and the presence of an AB doublet at δ_H_ 4.17 (*J =* 11.0 Hz) and 4.67 (*J =* 11.0 Hz) and a doublet vinyl proton at δ_H_ 5.91 (*J =* 1.5 Hz). From COSY spectrum measured in CDCl_3_, it was possible to establish thirteen proton sequences from H_2_-1 to H_2_-2, H_2_-6 to H_2_-7, H_2_-7 to H-8, H-8 to H-9 and H-14, H-9 to H_2_-11, H_2_-11 to H_2_-12, H-14 to H_2_-15, H-17 to H-20, and H-20 to H_2_-21 (Figure 1). The HMBC correlations of H_2_-19 to C-1, C-5, C-9 and C-10; H_3_-18 to C-12, C-13 and C-17; H_2_-2 to C-3; H-4 to C-2, C-6 and C-10; H_2_-15 to C-16 and C-17, permitted the connection of the carbon skeleton. The observed NOESY correlations between H_3_-18 and both H-20 and H-8, H-8 and H-6β, H-11β and H_2_-19, H-14 and both H-17 and H-9, H-6α and both H-4 and H-9 revealed the β-orientation of H-6β, H-8, H-11β, H_3_-18, H_2_-19, and H-20 and α-orientation of H-4, H-6α, H-9, H-14, and H-17 (Figure 2). On the basis of the above spectroscopic data, the structure of **2** was established as 19-acetoxypregna-4,20-dien-3-one.

Sclerosteroid L (**3**) was shown by HRESIMS to possess the molecular formula C_23_H_36_O_3_ (*m*/*z* 383.2559 [M + Na]^+^). The IR absorptions at 3568 and 1712 cm^−1^ suggested the presence of hydroxy and carbonyl groups. The ^13^C NMR and DEPT spectrum of **3** were similar to those of 5α-pregn-20-en-1α,3α-diol 3-acetate [18], with small differences observed in the A-ring. This was confirmed by the COSY correlations between H-1/H_2_-2, H_2_-2/H-3, and H-3/H_2_-4, revealing that **3** is an epimer of 5α-pregn-20-en-1α,3α-diol 3-acetate (Figure 1). The planar structure of **3** was further confirmed by analysis of ^1^H–^1^H COSY and HMBC correlations (Figure 1). The ^1^H–^1^H COSY correlations allowed the establishment of two additional spin systems from H-5 to H-8 and H-14 to H-17 through H-20 to H_2_-21. Key HMBC correlations of H_3_-19 to C-1, C-5, C-9 and C-10; H_3_-18 to C-12, C-13, C-14 and C-17; H-4 to and C-5; H-9 to C-8 and C-11; H_2_-12 to C-11 and C-14, permitted establishment of the planar structure of **3**. The observed NOESY correlations between H-20 and H_3_-18, H_3_-19 and H-2β, H_3_-18 and H-8, H-17 and H-14, H-14 and H-9, H-9 and H-1α, H-5 and H-3α, H-3α and H-2α, and H-2α and H-1α revealed the β-orientation of H-2β, H-8, H_3_-18, H_3_-19, and H-20 and α-orientation of H-1α, H-2α, H-3α, H-5, H-9, H-14, and H-17 (Figure 2). Analysis of the coupling constants of H-1 (δ_H_ 3.51, 1H, td, *J* = 11.5, 5.0 Hz) and H-3 (δ_H_ 4.70, 1H, tt, *J* = 11.5, 5.0 Hz) confirming the β-orientations of 1-OH and 3-OH. Therefore, the structure of **3** could be established unambiguously as 5α-pregn-20-en-1β,3β-diol 3-acetate.

The molecular formula of sclerosteroid M (**4**) was found to be C_31_H_4__8_O_8_, as established HRESIMS, ^13^C NMR, and DEPT data, indicating eight degrees of unsaturation. The ^1^H and ^13^C NMR spectra of **4** displayed the signals of a vinyl group (δ_H_ 4.95, 1H, br d, *J* = 18.0 Hz; 4.96, 1H, br d, *J* = 10.0 Hz; 5.74, 1H, ddd, *J* = 18.0, 10.0, 8.0 Hz; δ_C_ 114.5, 139.8), two ester carbonyl (δ_C_ 171.3, 171.5), and two acetate methyl groups (δ_H_ 2.06, 3H, s; 2.13, 3H, s; δ_C_ 21.1, 21.2). Therefore, 4 possesses five rings. The ^1^H and ^13^C NMR spectroscopic data of **4** were similar to those of sclerosteroid A [7], except for the appearing of six additional carbon signals at δ_C_ 94.6 (CH), 72.0 (CH), 68.6 (CH), 72.3 (CH), 65.3 (CH), and 16.1 (CH_3_), an anomeric proton signal at δ_H_ 5.13 (1H, d, *J* = 4.0 Hz), as well as a methyl doublet at δ_H_ 1.28, suggesting the presence of a 6′-deoxyhexose unit. This hexose appeared to be the C-2′ monoacetate derivative of fucopyranose by comparison of ^1^H and ^13^C NMR data with those reported previously [7] and on the basis of the results of ^1^H–^1^H COSY, HMBC, and NOESY experiments, in particular the HMBC correlation from H-2′ (δ_H_ 4.86) to the acetate carbonyl carbon (δ_C_ 171.5) (Figure 1). The sugar moiety was found to be connected with C-3 of the aglycon by HMBC correlation of H-1′ and C-3. The anomeric proton H-1′ (δ_H_ 5.13) has a small coupling constant, indicating the equatorial orientation of this proton. The relative configuration of the aglycon of **4** was further determined by NOESY experiment (Figure 2). On the basis of the above analysis, the structure of **4** was established as 3β-(2′-*O*-acetyl-α-l-fucopyranosyloxy)-5α-pregn-20-en-19-ol 19-acetate.

Sclerosteroid N (**5**) had a molecular formula of C_29_H_44_O_7_ as established by HRESIMS. The ^1^H and ^13^C NMR spectroscopic data of **5** were similar to those of **4**, except for the replacement of the C-10 acetoxymethyl group in **4** by an aldehyde (δ_H_ 10.0, 1H, s; δ_C_ 208.5) in **5** (Table 2). The relative configuration and connection of the aglycon and sugar residue of **5** were further determined by ^1^H–^1^H COSY, HMBC, and NOESY experiments (Figure 1, Figure 2). Thus, the structure of **5** was assigned as 3β-(2′-*O*-acetyl-α-l-fucopyranosyloxy)-5α-pregna-20-en-19-al.

**Table 2:** ^1^H and ^13^C NMR spectroscopic data of **4**–**6**.

Position		4		5		6	
		δ_H_ (*J* in Hz) ^a^	δ_C_ (mult.) ^b^	δ_H_ (*J* in Hz) ^c^	δ_C_ (mult.) ^d^	δ_H_ (*J* in Hz) ^c^	δ_C_ (mult.) ^d^
1	α	0.91 m	31.9, CH_2_^e^	0.93 m	31.0, CH_2_		165.4, C
	β	2.23 m		2.40 dt (14.0, 4.0)			
2	α	1.83 m	29.3, CH_2_	1.40 m	30.3, CH_2_	5.81 s	116.6, CH
	β	1.44 m		1.87 m			
3		3.53 m	76.7, CH	3.50 m	76.7, CH		157.0, C
4	α	1.62 m	34.4, CH_2_	1.71 m	35.9, CH_2_	2.22 d (4.4) ^f^	35.0, CH_2_
	β	1.33 m		1.23 m		2.27 m	
5		1.25 m	44.8, CH	1.36 m	43.3, CH	4.46 m	75.7, CH
6	α	1.27 m	28.3, CH_2_	1.53 m	28.3, CH_2_	1.99 s	23.0, CH_3_
	β			1.91 m			
7	α	0.92 m	32.0, CH_2_	1.07 m	32.0, CH_2_		
	β	1.75 m		1.90 m			
8		1.50 m	35.9, CH	1.43 m	37.0, CH		
9		0.75 m	54.5, CH	0.95 m	52.8, CH		
10			38.1, C		51.8, C		
11	α	1.65 m	21.8, CH_2_	1.26 m	21.4, CH_2_		
	β	1.35 m		1.69 m			
12	α	0.97 m	37.8, CH_2_	0.98 m	37.3, CH_2_		
	β	1.68 m		1.66 m			
13			43.6, C		43.4, C		
14		1.02 m	55.9, CH	0.93 m	55.7, CH		
15	α	1.68 m	24.7, CH_2_	1.14 m	24.6, CH_2_		
	β	1.18 m		1.66 m			
16	α	1.79 m	27.2, CH_2_	1.50 m	27.1, CH_2_		
	β	1.56 m		1.76 m			
17		1.95 dt (18.0,8.0) ^f^	55.3, CH	1.93 m	55.3, CH		
18		0.57 s	13.0, CH_3_	0.52 s	12.8, CH_3_		
19		4.20 d (12.0)	62.8, CH_2_	10.0 s	208.5, CH		
		4.34 d (12.0)					
20		5.74 ddd (18.0, 10.0, 8.0)	139.8, CH	5.72 ddd (18.0, 10.0, 8.0)	139.5, CH		
21		4.95 br d (18.0)	114.5, CH_2_	4.96 br d (18.0)	114.7, CH_2_		
		4.96 br d (10.0)		4.97 br d (10.0)			
1′		5.13 d (4.0)	94.6, CH	5.12 d (3.6)	94.8, CH	1.52 m	42.3, CH_2_
						1.68 m	
2′		4.86 dd (10.5, 4.0)	72.0, CH	4.84 dd (10.0, 3.6)	72.0, CH	2.66 m	28.9, CH
3′		4.00 dd (10.5, 3.5)	68.6, CH	3.98 dd (10.0, 3.2)	68.5, CH	1.34 m	36.9, CH_2_
4′		3.81 d (3.5)	72.3, CH	3.80 d (3.2)	72.3, CH	1.20 m	24.2, CH_2_
						1.34 m	
5′		4.09 q (6.5)	65.3, CH	4.06 q (6.8)	65.4, CH	1.08 m	37.2, CH_2_
6′		1.28 d (6.5)	16.1, CH_3_	1.27 d (6.8)	16.1, CH_3_	1.38 m	32.7, CH
7′						1.08 m	37.3, CH_2_
						1.26 m	
8′						1.24 m	21.3, CH_2_
9′						1.70 m	41.0, CH_2_
10′							82.5, C
11′						1.42 s	26.1, CH_3_
12′						1.42 s	26.1, CH_3_
13′						0.85 d (6.4)	19.6, CH_3_
14′						0.93 d (6.0)	19.9, CH_3_
OAc		2.06 s	21.1, CH_3_	2.12 s	21.1, CH_3_	1.97 s	22.5, CH_3_
		2.13 s	21.2, CH_3_		171.5, C		170.5, C
			171.3, C				
			171.5, C				

^a^ Spectra recorded at 500 MHz in CDCl_3_; ^b^ Spectra recorded at 125 MHz in CDCl_3_; ^c^ Spectra recorded at 400 MHz in CDCl_3_; ^d^ Spectra recorded at 100 MHz in CDCl_3_; ^e^ Deduced from DEPT; ^f^ *J* values (Hz) in parentheses.

3-Methyl-5-(10′-acetoxy-2′,6′,10′-trimethylundecyl)-2-penten-5-olide (**6**) was obtained as colorless oil with [α]_D_^25^ −22 (*c* 0.17, CHCl_3_). The HRESIMS of **6** exhibited a [M + Na]^+^ peak at *m/z* 389.2666, and established a molecular formula of C_22_H_38_O_4_, implying four degrees of unsaturation. Above information and the UV absorption at 211 nm and the IR absorption at 1729 cm^−1^ suggested the presence of an α,β-unsaturated δ-lactone [19]. The ^13^C NMR and DEPT spectroscopic data (Table 2) showed 22 carbon signals, including six methyls, eight sp^3^ methylenes, three sp^3^ methines, one sp^2^ methine, three sp^2^ quaternary and one sp^3^ quaternary carbons. The signals in the ^1^H NMR spectrum at δ_H_ (5.81, 1H, s), 2.22 (1H, d, *J* = 4.4 Hz), 2.27 (1H, m), 4.46 (1H, m), and 1.99 (3H, s) verified the presence of a subunit of 3-methyl-2-penten-5-olide [20,21]. The planar structure and all of the ^1^H and ^13^C chemical shifts of **6** were elucidated by 2D NMR experiments, in particular the ^1^H–^1^H COSY and HMBC experiments (Figure 1). The HMBC correlations from H_2_-5 to C-4 and C-1′; H_3_-11′ to C-9′ and C-10′; H_3_-12′ to C-10′; H_3_-13′ to C-5′ and C-6′; H_3_-14′ to C-1′, C-2′ and C-3′; and H_3_-6 to C-3 and C-4. The structure of **6** thus was established and was found to be a α,β-unsaturated δ-lactone, 3-methyl-5-(10′-acetoxy-2′,6′,10′-trimethylundecyl)-2-penten-5-olide, which is a new chemotype of diterpene.

**Figure 3:**
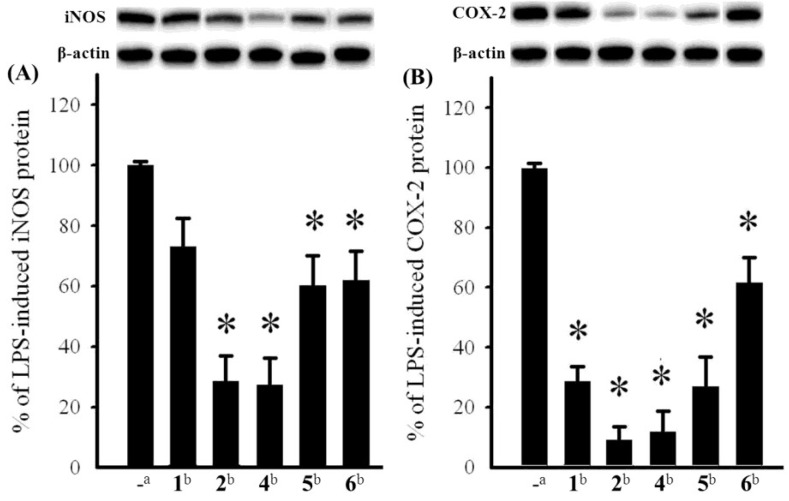
Effect of isolates (10 μM) from *S. gracillimum* on the lipopolysaccharide (LPS)-induced pro-inflammatory iNOS and on COX-2 protein expression of RAW264.7 macrophage cells by immunoblot analysis. (**A**) Immunoblots iNOS and β-actin; (**B**) Immunoblots COX-2 and β-actin. The values are means ± SEM (*n* = 6). The relative intensity of the LPS alone stimulated group was taken as 100%. * Significantly different from LPS alone stimulated group (* *p* < 0.05). ^a^ stimulated with LPS; ^b^ stimulated with LPS in the presence of **1**, **2**, and **4**–**6** (10 μM).

**Chart 2:**
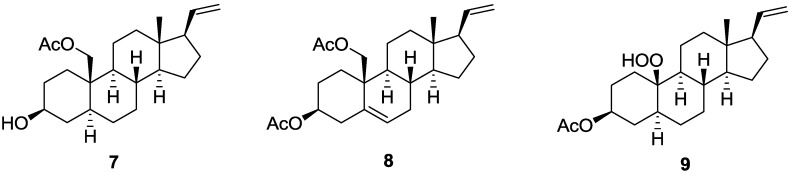
Structures of known anti-inflammatory compounds **7**–**9**.

The cytotoxicity of compounds **3**–**5** against the proliferation of a limited panel of cancer cell lines, including HepG2, A549, and MDA-MB-231 carcinoma cell lines was evaluated. The results showed that compound **4** exhibited moderate cytotoxicity against HepG2, A549, and MDA-MB-231 with IC_50_ values of 23.3, 21.9, and 24.3 μM, respectively. The anti-inflammatory activities of **1**, **2**, and **4**–**6** against the accumulation of pro-inflammatory iNOS and COX-2 proteins in RAW264.7 macrophage cells stimulated with LPS, were also evaluated using immunoblot analysis (Figure 3). At a concentration of 10 μM, compounds **2**, and **4** significantly reduced the levels of iNOS protein to 28.4 ± 8.4%, and 27.2 ± 9.0%, respectively, relative to control cells stimulated with LPS only. Meanwhile, compounds **1**, **5**, and **6** moderately reduced iNOS level to 72.8 ± 9.5%, 60.3 ± 9.7%, and 61.8 ± 9.8%, respectively. At the same concentration, compounds **1**, **2**, and **4**–**6** also could reduce COX-2 expression to 28.4 ± 4.9%, 9.0 ± 4.4%, 11.8 ± 6.8%, 26.6 ± 10.0%, and 61.7 ± 8.3%, respectively. These results indicated that compounds **2** and **4** might become the effective anti-inflammatory agents as they can potently inhibit the expression of both iNOS and COX-2 proteins in LPS-induced macrophage cells. Compounds **1** and **5** might also be useful anti-inflammatory compounds as they could effectively reduce COX-2 expression, too. Thus, soft coral *S. gracillimum* is an important source of anti-inflammatory agents, as except **1**, **2**, **4** and **5**, effective anti-inflammatory compounds sclerosteroids A (**7**), B (**8**), and E (**9**) (Chart 2) were also discovered from this organism by our previous investigation [7]. The exhibited anti-inflammatory activity for sclerosteroids A, B, E, J, K, M, and N revealed that the oxidation at C-19 in pregnanes could enhance anti-inflammatory activity.

## 3. Experimental Section

### 3.1. General Experimental Procedures

Melting point was determined using a Fisher-Johns melting point apparatus. Optical rotations were measured with a JASCO P-1020 polarimeter. Ultraviolet spectrum was recorded on a JASCO V-650 spectrophotometer. IR spectrum was recorded on a JASCO FT/IR-4100 spectrophotometer. The NMR spectra were recorded on a Varian MR-400 FT-NMR or Varian Unity INOVA 500 FT-NMR instrument at 400 MHz or 500 MHz for ^1^H (referenced to TMS, δ_H_7.27 ppm for CDCl_3_) and 100 or 125 MHz for ^13^C (referenced to δ_C_ 77.0 for CDCl_3_). ESIMS and HRESIMS were recorded by ESI FT-MS on a Bruker APEX II mass spectrometer. Silica gel 60 (Merck, 230–400 mesh) was used for column chromatography. Precoated silica gel plates (Merck, Kieselgel 60 F_254_, 0.25 mm) and precoated RP-18 F_254__S_ plates (Merck, 1.05560) were used for TLC analyses. High-performance liquid chromatography (HPLC) was performed on a Hitachi L-7100 pump equipped with a Hitachi L-7400 UV detector and a semi-preparative reversed-phase column (YMC-Pack Pro C18, 5 μm, 250 × 10 mm).

### 3.2. Animal Material

The soft coral *Scleronephthya gracillimum* was collected at Green Island, Taiwan, in January, 2008, at a depth of 10 m, and was stored in a freezer until extraction. This soft coral was identified by one of the authors (C.-F.D.). A voucher specimen (NSYSU-SG001) was deposited in the Department of Marine Biotechnology and Resources, National Sun Yat-sen University.

### 3.3. Extraction and Isolation

The frozen bodies of *S.* *gracillimum* (1.3 kg, fresh wet) were minced and extracted with EtOH (3 L × 2). The organic extract was concentrated to an aqueous suspension which was further partitioned between EtOAc and H_2_O. The EtOAc extract (9.6 g) was fractionated by open column chromatography on silica gel using *n*-hexane–EtOAc and EtOAc–MeOH mixtures of increasing polarity to yield 40 fractions. Fraction 13, eluting with *n*-hexane–EtOAc (4:1), was further separated by silica gel column chromatography (*n*-hexane–EtOAc, 10:1) and followed by reversed-phase HPLC (MeOH–H_2_O, 85:15) to afford **1** (1.0 mg), **2** (1.0 mg), and **6** (1.7 mg). Fraction 14, eluting with *n*-hexane–EtOAc (2:1), was further separated by silica gel column chromatography (*n*-hexane–EtOAc, 10:1) and followed by reversed-phase HPLC (MeOH–H_2_O, 85:15) to afford **3** (0.9 mg). Fraction 18, eluting with *n*-hexane–EtOAc (1:6), was rechromatographed over a Sephadex LH-20 column using acetone as the mobile phase to afford six subfractions (A1–A6). Subfractions A2 was separated by reversed-phase HPLC (CH_3_CN–H_2_O, 75:25) to afford compounds **4** (2.0 mg), and **5** (2.5 mg).

Sclerosteroid J (**1**): colorless oil; [α]_D_^25^ −60 (*c* 0.1, CHCl_3_); IR (neat) ν_max_ 2926, 2865, 1739, 1576, 1452, 1236, and 1051 cm^−1^; ^13^C and ^1^H NMR data, see Table 1; ESIMS *m/z* 427 [M + Na]^+^; HRESIMS *m/z* 427.2827 [M + Na]^+^ (calcd for C_2__5_H_40_O_4_Na, 427.2824).

Sclerosteroid K (**2**): colorless oil; [α]_D_^25^ −41 (*c* 0.1, CHCl_3_); IR (neat) ν_max_ 2926, 2870, 1744, 1677, 1233, and 1036 cm^−1^; UV (MeOH) λ_max_ 239 (log ε = 4.2); ^13^C and ^1^H NMR data, see Table 1; ESIMS *m/z* 379 [M + Na]^+^; HRESIMS *m/z* 379.2251 [M + Na]^+^ (calcd for C_2__3_H_3__2_O_3_Na, 379.2249).

Sclerosteroid L (**3**): white powder; mp 150–152 °C; [α]_D_^25^ −18 (*c* 0.07, CHCl_3_); IR (neat) ν_max_ 3568, 2922, 2870, 1712, 1244, and 1025 cm^−1^; ^13^C and ^1^H NMR data, see Table 1; ESIMS *m/z* 383 [M + Na]^+^; HRESIMS *m/z* 383.2559 [M + Na]^+^ (calcd for C_2__3_H_3__6_O_3_Na, 383.2562).

Sclerosteroid M (**4**): yellow oil; [α]_D_^25^ −60 (*c* 0.1, CHCl_3_); IR (neat) ν_max_ 3448, 2926, 2868, 1739, 1369, 1241, and 1034 cm^−1^; ^13^C and ^1^H NMR data, see Table 2; ESIMS *m/z* 571 [M + Na]^+^; HRESIMS *m/z* 571.3249 [M + Na]^+^ (calcd for C_31_H_48_O_8_Na, 571.3247).

Sclerosteroid N (**5**): colorless oil; [α]_D_^25^ −24 (*c* 0.25, CHCl_3_); IR (neat) ν_max_ 3390, 2926, 2870, 1739, 1374, 1250, and 1036 cm^−1^; ^13^C and ^1^H NMR data, see Table 2; ESIMS *m/z* 527 [M + Na]^+^ ; HRESIMS *m/z* 527.2989 [M + Na]^+^ (calcd for C_2__9_H_44_O_7_Na, 527.2985).

3-Methyl-5-(10′-acetoxy-2′,6′,10′-trimethylundecyl)-2-penten-5-olide (**6**): colorless oil; [α]_D_^25^ −22 (*c* 0.17, CHCl_3_); IR (neat) ν_max_ 2926, 2865, 1729, 1363, 1250, and 1014 cm^−1^; UV (MeOH) λ_max_ 211 (log ε = 4.0); ^13^C and ^1^H NMR data, see Table 2; ESIMS *m/z* 389 [M + Na]^+^ ; HRESIMS *m/z* 389.2666 [M + Na]^+^ (calcd for C_2__2_H_38_O_4_Na, 389.2668).

### 3.4. Cytotoxicity Testing

Cell lines were purchased from the American Type Culture Collection (ATCC). Cytotoxicity assays of compounds **1**–**6** were performed using the MTT [3-(4,5-dimethylthiazol-2-yl)-2,5-diphenyl-tetrazolium bromide] colorimetric method [22,23].

### 3.5. *In Vitro* Anti-Inflammatory Assay

Macrophage (RAW264.7) cell line was purchased from ATCC. *In vitro* anti-inflammatory activities of tested compounds were measured by examining the inhibition of lipopolysaccharide (LPS) induced upregulation of iNOS (inducible nitric oxide synthase) and COX-2 (cyclooxygenase-2) proteins in macrophages cells using western blotting analysis [24,25].

## 4. Conclusions

Our present investigation again demonstrated that the Formosan soft coral *Scleronephthya gracillimum* could be a source of anti-inflammatory natural products. Anti-inflammatory activity assay revealed that compounds **1**, **2** and **4**–**6**, in particular **2** and **4**, and the previously discovered pregnanes sclerosteroids A, B, and E, deserve further study for therapeutic potential against inflammation-related diseases.

## Supplementary Files

Supplementary File 1 Supplementary Information (PDF, 1469 KB)
