# Supplementary material for: Cytotoxic and Anti-Inflammatory Metabolites from the Soft Coral Scleronephthya gracillimum
Source: Mar Drugs. 2013 May 29;11(6):1853–65. doi: 10.3390/md11061853 (PMC3721209; doi:10.3390/md11061853)

Supplementary Information

**Figure S1.**	^{1}H NMR spectrum of **1** in $CDCl_3$ at 500 MHz.	2
**Figure S2**.	^{13}C NMR spectrum of **1** in $CDCl_3$ at 125 MHz.	3
**Figure S3.**	HRESI spectrum of **1**.	4
**Figure S4**.	^{1}H NMR spectrum of **2** in $CDCl_3$ at 500 MHz.	5
**Figure S5.**	^{13}C NMR spectrum of **2** in $CDCl_3$ at 125 MHz.	6
**Figure S6.**	HRESI spectrum of **2**.	7
**Figure S7.**	^{1}H NMR spectrum of **3** in $CDCl_3$ at 400 MHz.	8
**Figure S8.**	^{13}C NMR spectrum of **3** in $CDCl_3$ at 100 MHz.	9
**Figure S9.**	HRESI spectrum of **3**.	10
**Figure S10.**	^{1}H NMR spectrum of **4** in $CDCl_3$ at 500 MHz.	11
**Figure S11.**	^{13}C NMR spectrum of **4** in $CDCl_3$ at 125 MHz.	12
**Figure S12.**	HRESI spectrum of **4**.	13
**Figure S13.**	^{1}H NMR spectrum of **5** in $CDCl_3$ at 500 MHz.	14
**Figure S14.**	^{13}C NMR spectrum of **5** in $CDCl_3$ at 125 MHz.	15
**Figure S15.**	HRESI spectrum of **5**.	16
**Figure S16.**	^{1}H NMR spectrum of **6** in $CDCl_3$ at 400 MHz.	17
**Figure S17.**	^{13}C NMR spectrum of **6** in $CDCl_3$ at 100 MHz.	18
**Figure S18.**	HRESI spectrum of **6**.	19

Figure S1. ^{1}H NMR spectrum of **1** in $CDCl_3$ at 500 MHz.

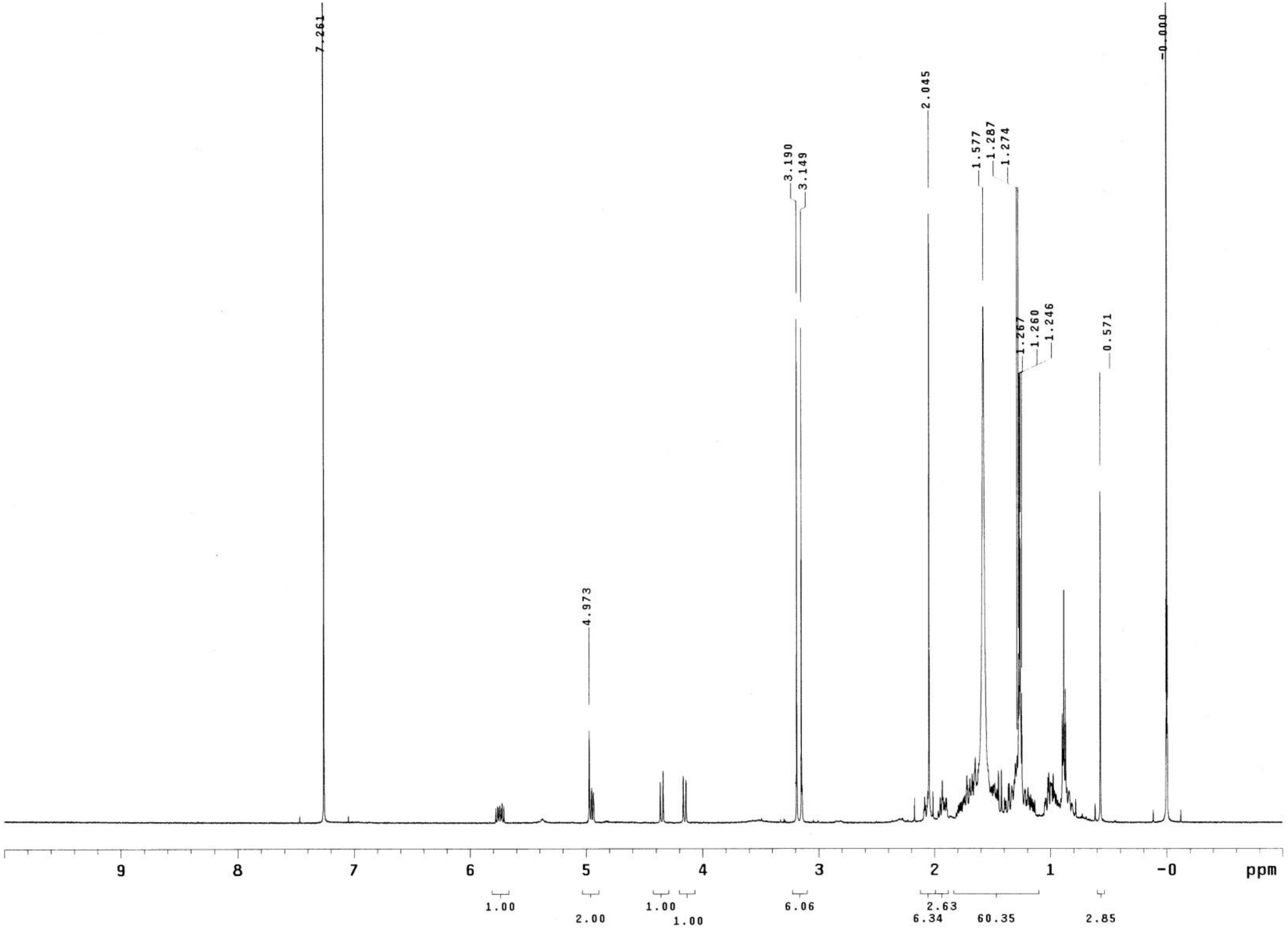

Figure S2. ^{13}C NMR spectrum of **1** in $CDCl_3$ at 125 MHz.

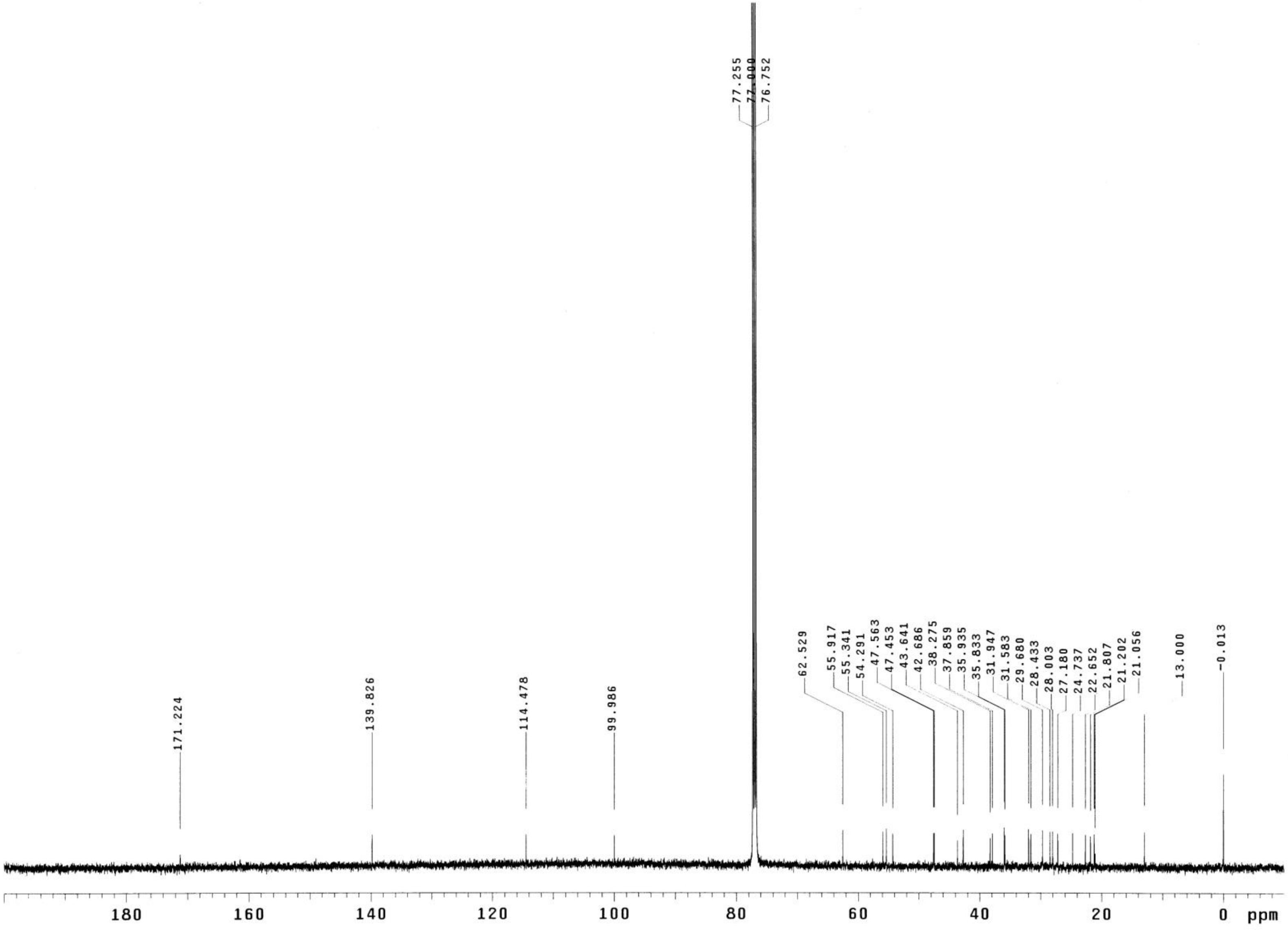

Figure S3. HRESIMS spectrum of **1**.

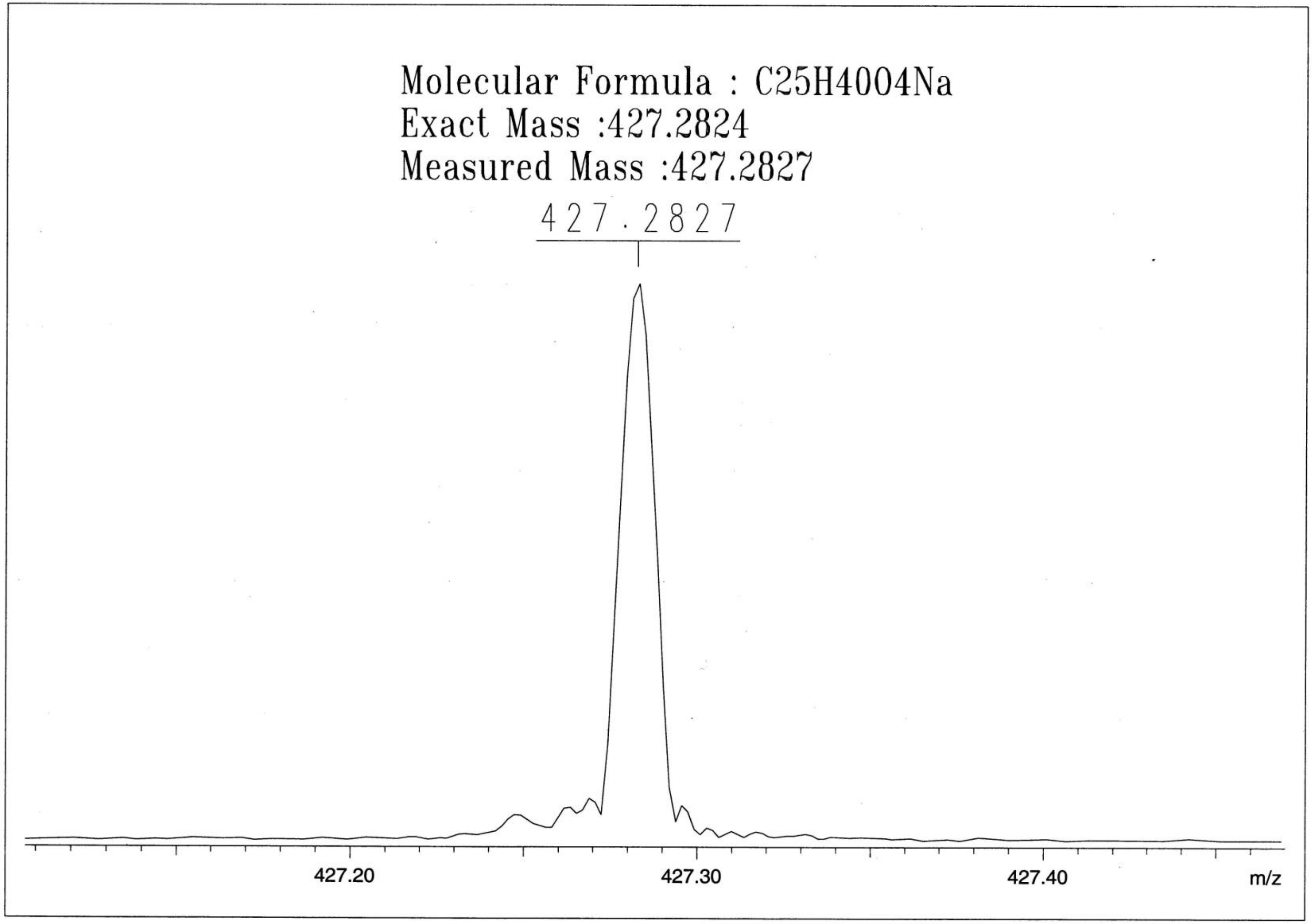

Figure S4. ^{1}H NMR spectrum of **2** in $CDCl_3$ at 500 MHz.

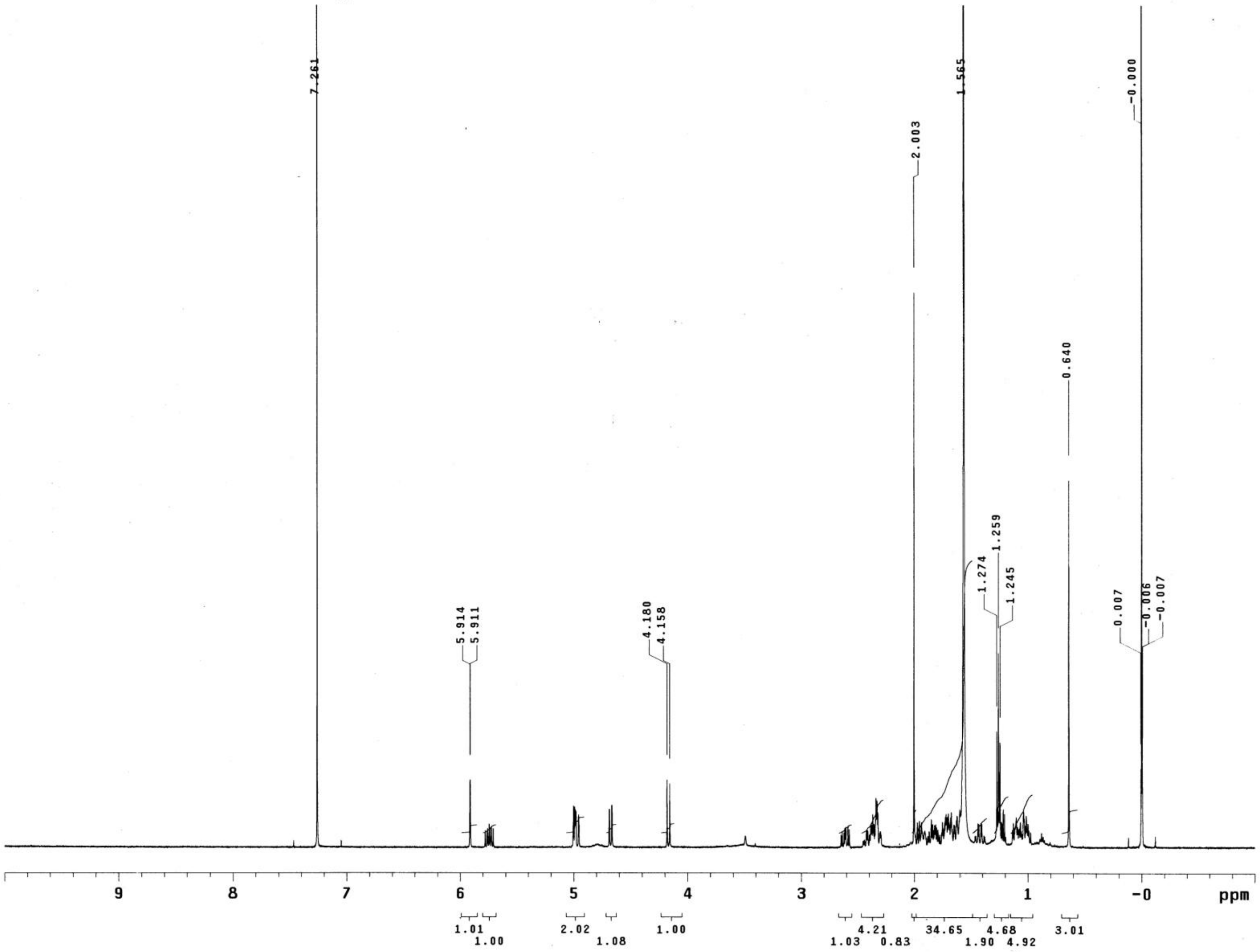

Figure S5. ^{13}C NMR spectrum of **2** in $CDCl_3$ at 125 MHz.

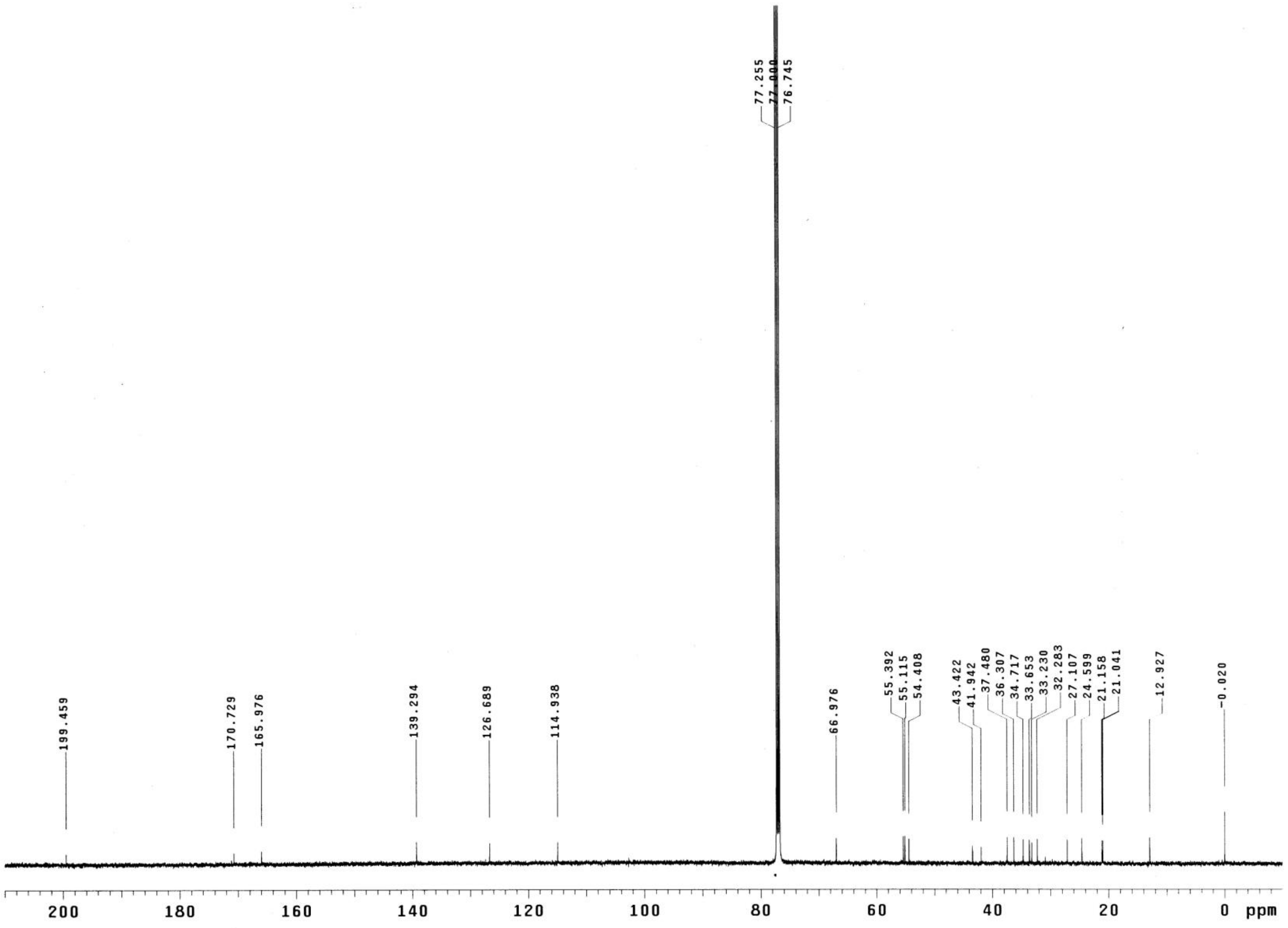

Figure S6. HRESIMS spectrum of **2**.

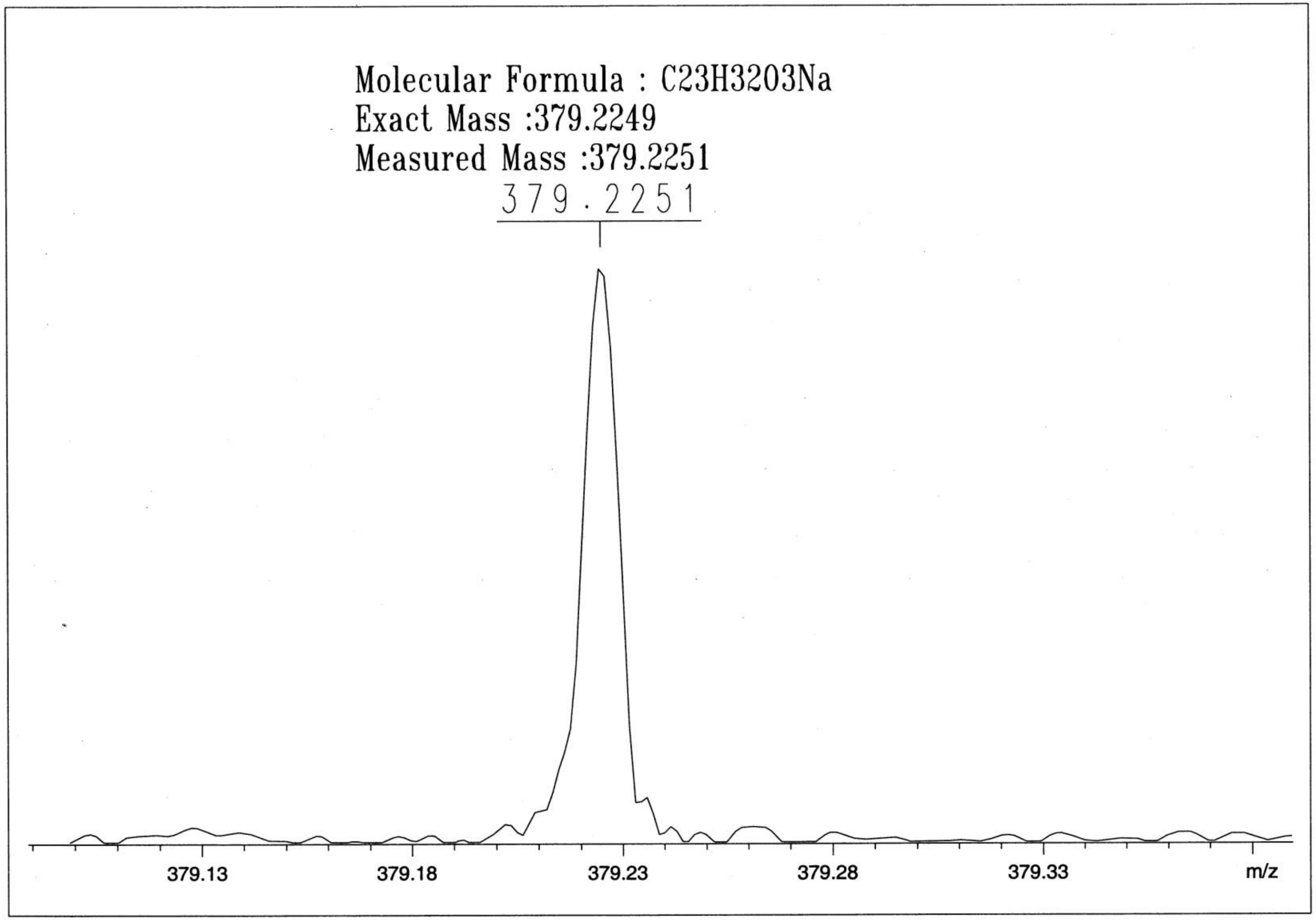

Figure S7. ^{1}H NMR spectrum of **3** in $CDCl_3$ at 500 MHz.

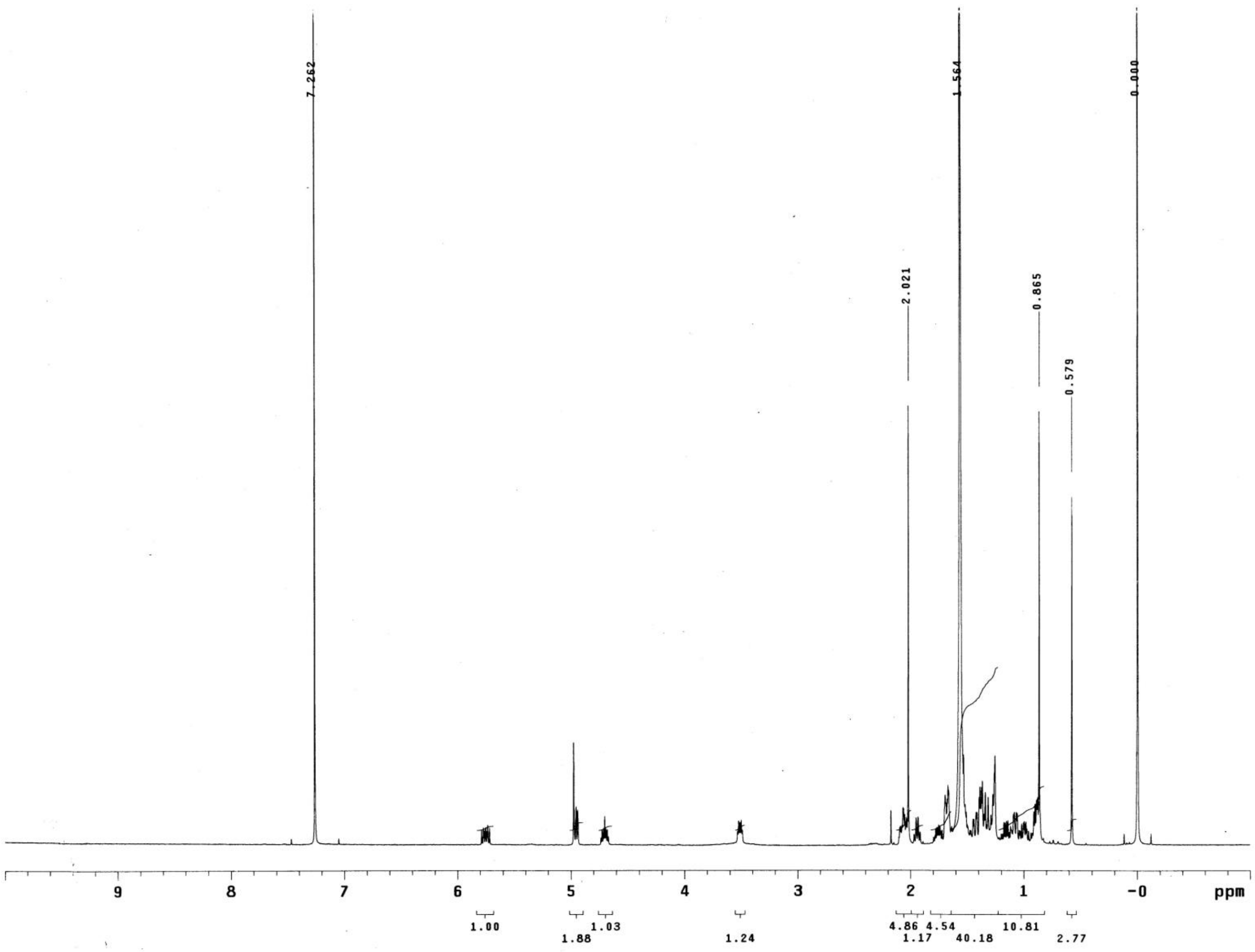

Figure S8. ^{13}C NMR spectrum of **3** in $CDCl_3$ at 125 MHz.

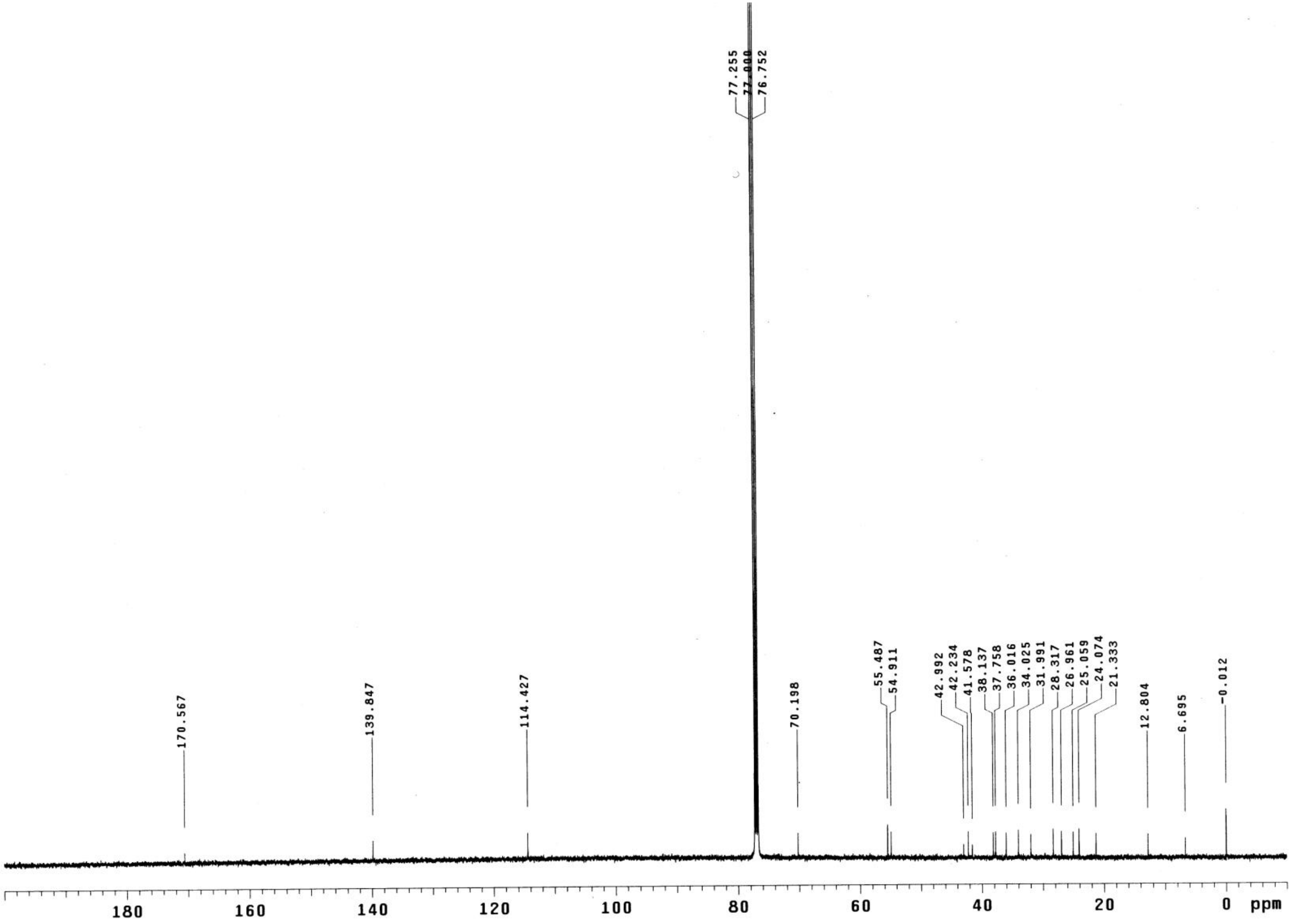

Figure S9. HRESIMS spectrum of **3**.

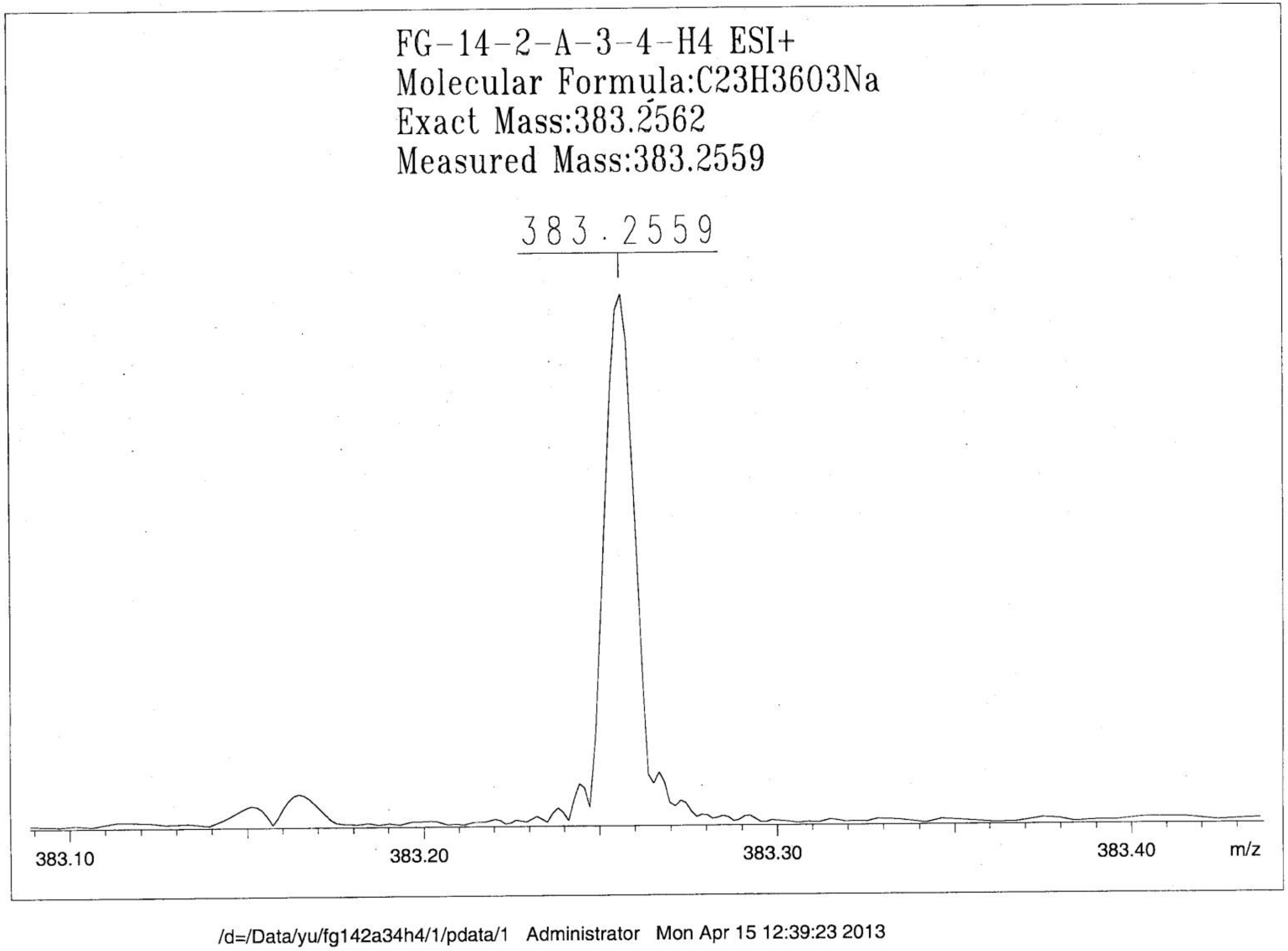

Figure S10. ^{1}H NMR spectrum of **4** in $CDCl_3$ at 500 MHz.

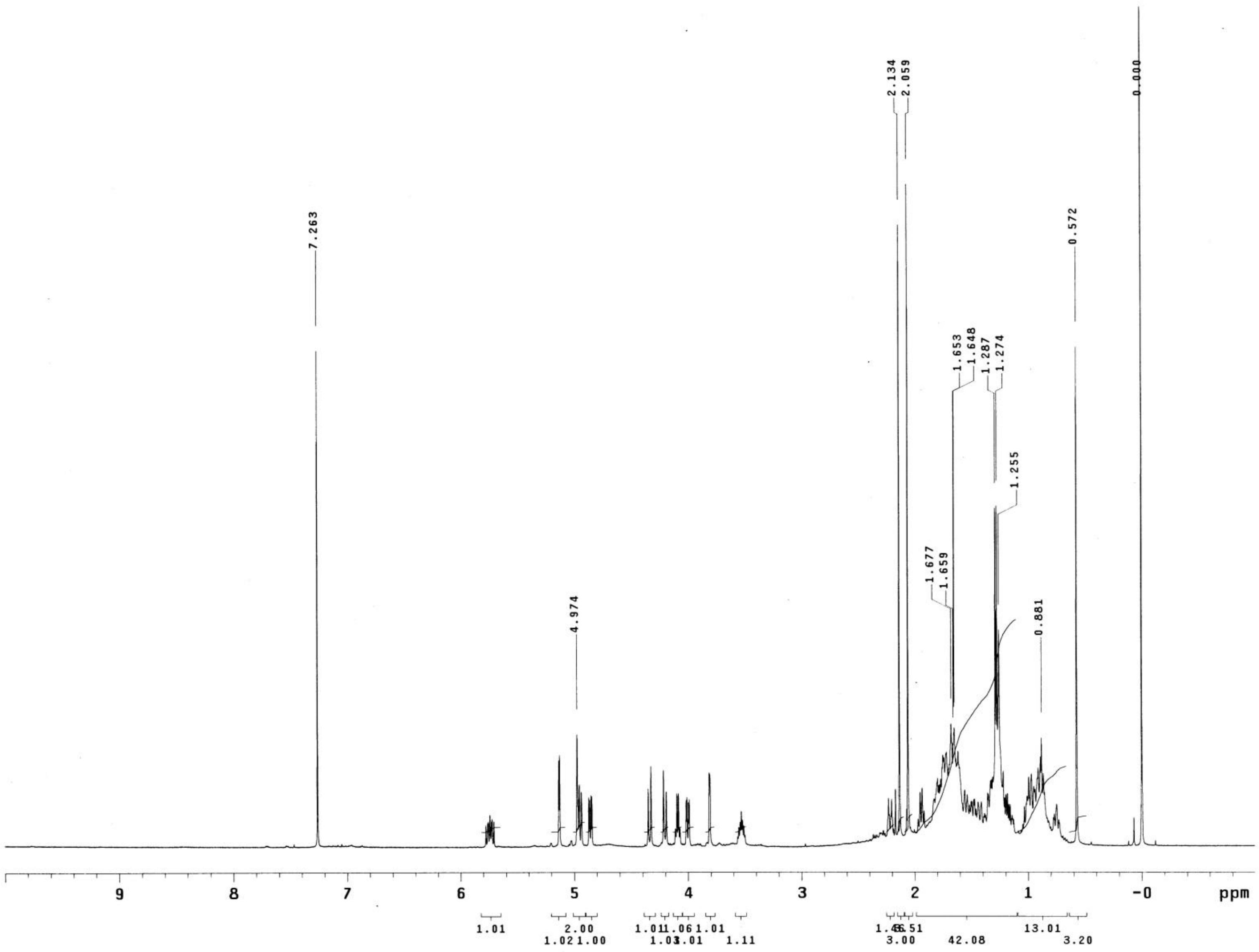

Figure S11. ^{13}C NMR spectrum of **4** in $CDCl_3$ at 125 MHz.

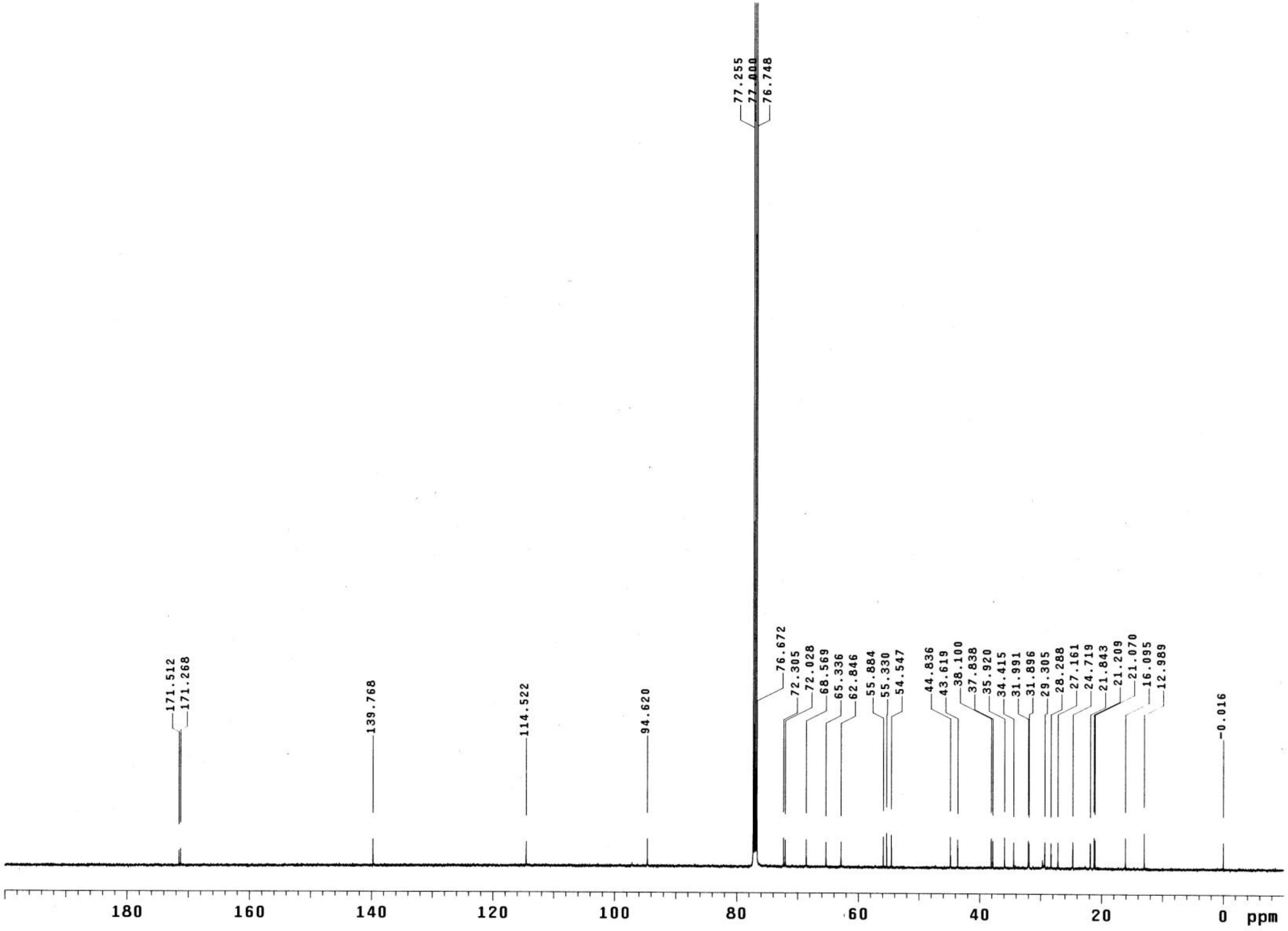

Figure S12. HRESIMS spectrum of **4**.

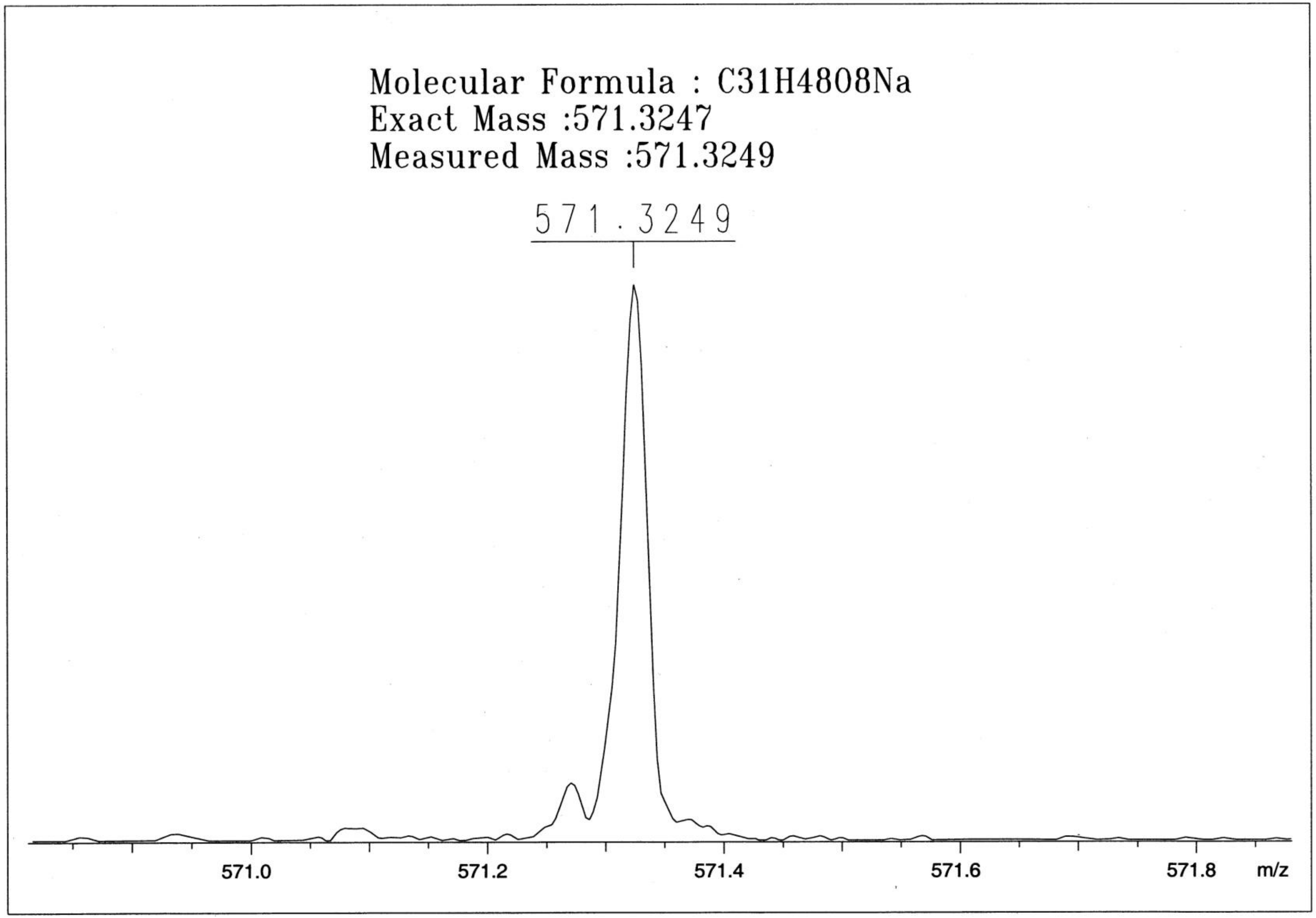

Figure S13. ^{1}H NMR spectrum of **5** in $CDCl_3$ at 400 MHz.

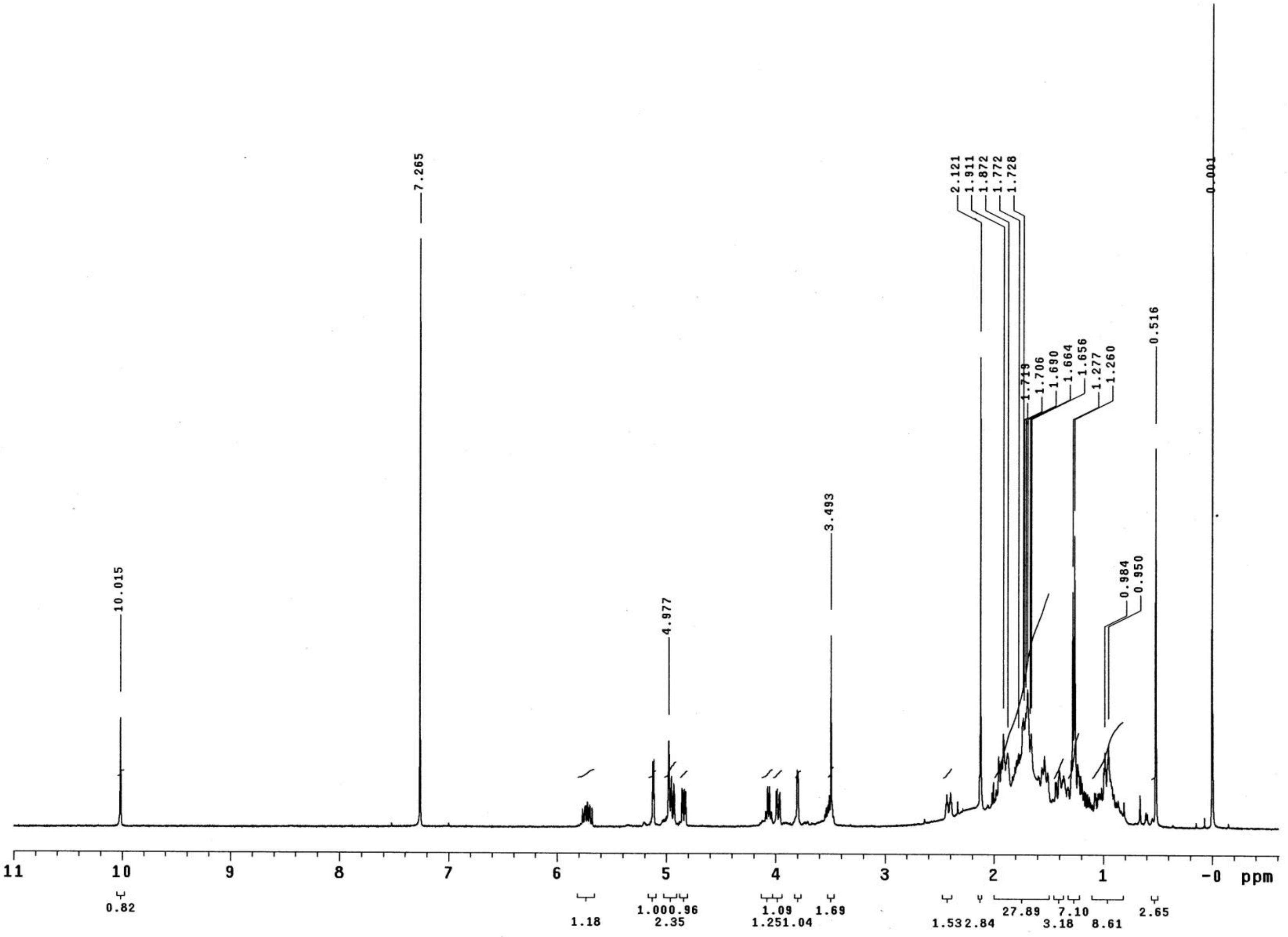

Figure S14. ^{13}C NMR spectrum of **5** in $CDCl_3$ at 100 MHz.

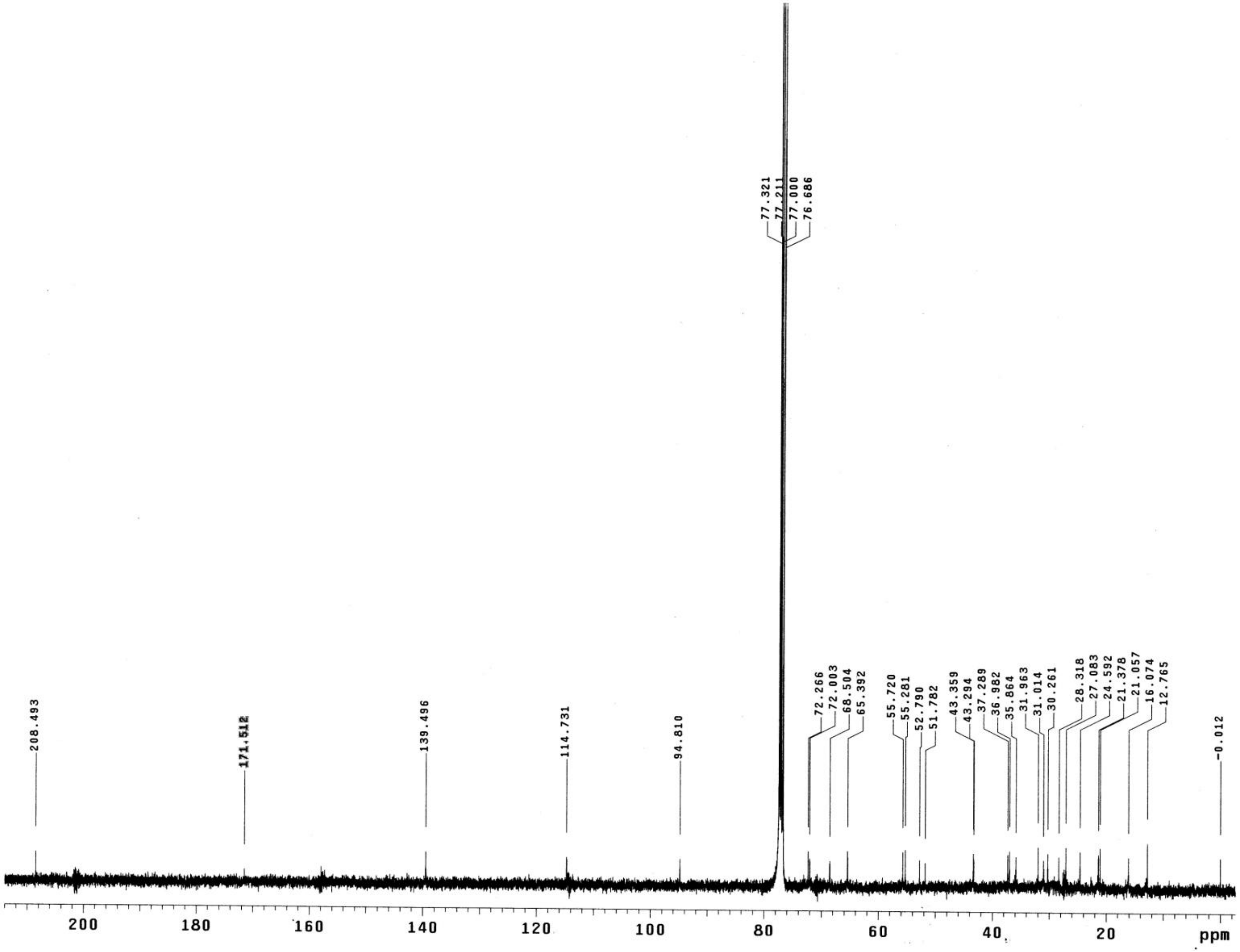

Figure S15. HRESIMS spectrum of **5**.

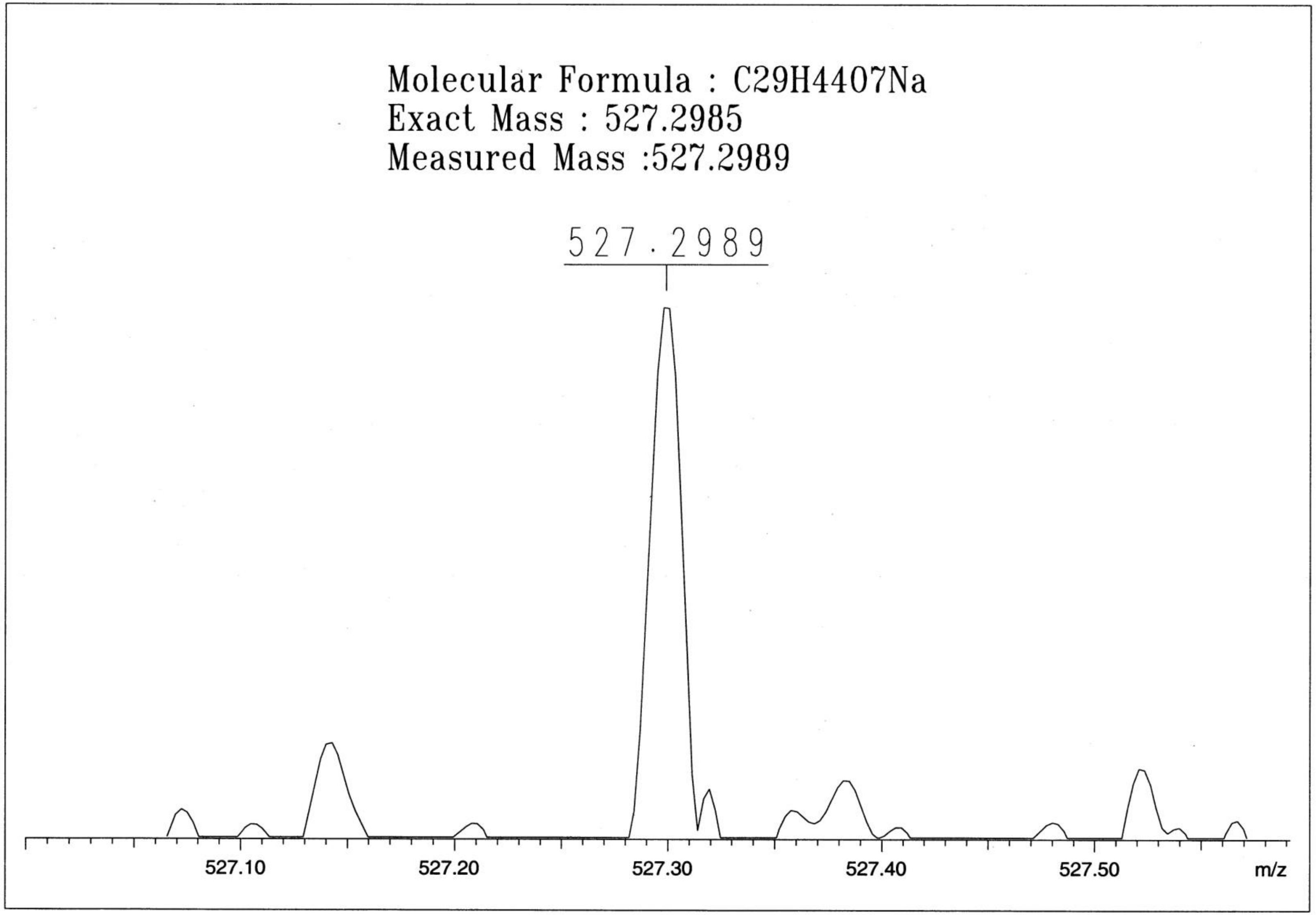

Figure S16. ^{1}H NMR spectrum of **6** in $CDCl_3$ at 400 MHz.

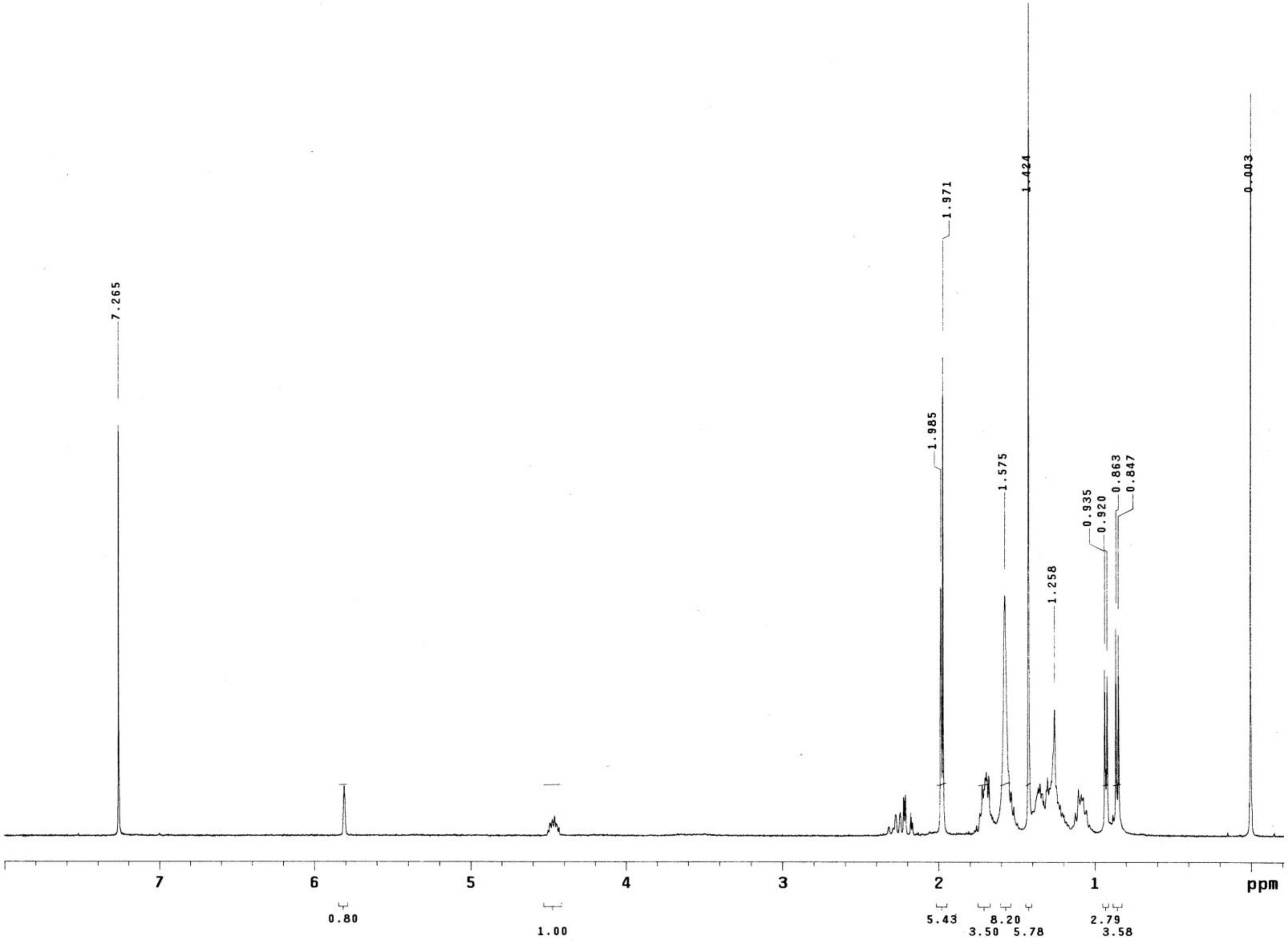

Figure S17. ^{13}C NMR spectrum of **6** in $CDCl_3$ at 100 MHz.

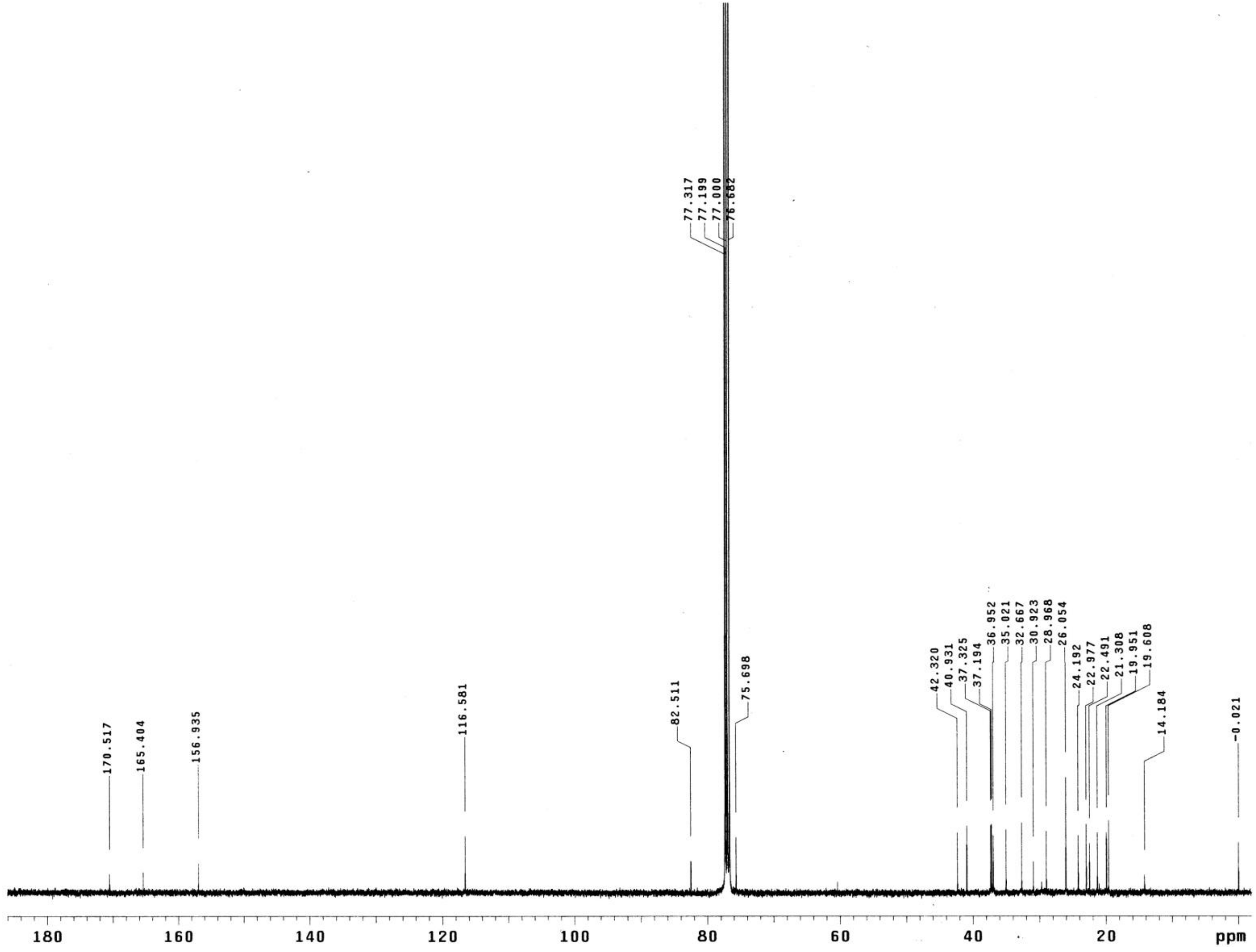

Figure S18. HRESIMS spectrum of **6**.

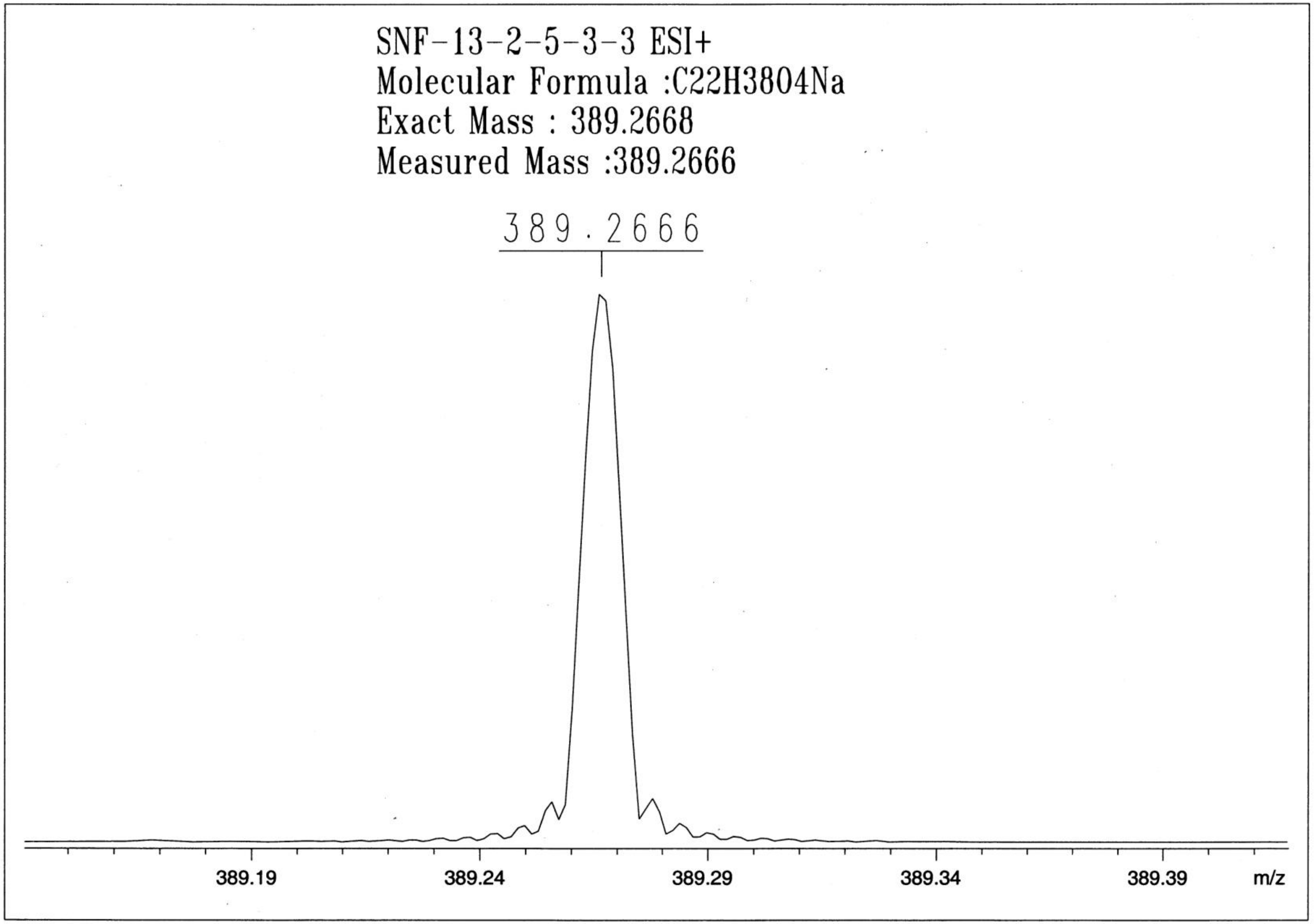

Supplement: Supplementary File 1 — Supplementary Information (PDF, 1469 KB) [file marinedrugs-11-01853-s001.pdf]
